# Supplementary material for: Caspase-1-driven neutrophil pyroptosis and its role in host susceptibility to Pseudomonas aeruginosa
Source: PLoS Pathog. 2022 Jul 18;18(7):e1010305. doi: 10.1371/journal.ppat.1010305 (PMC9345480; doi:10.1371/journal.ppat.1010305)
Supplement: S1 Table — (DOCX) [file ppat.1010305.s001.docx]

**S1 Table**

References and working concentrations of Reagents and Tools used in this study are listed.

| REAGENT or RESSOURCE | SOURCE | IDENTIFIER |
| --- | --- | --- |
| Antibodies |  |  |
| Anti- mouse Caspase 1, 1: 1000 | AdipoGen | AG20B-0042 |
| Anti-Ly6G APC-Vio770, 1:50 | Miltenyi-Biotec | **130-119-126** |
| Anti- mouse Gasdermin D, 1: 1000 | Abcam | AB209845 |
| Anti- mouse IL-1beta, 1: 1000 | R&D Systems | AF-401-NA |
| Anti-MPO , 1: 1000 | R&D Systems | AF3667 |
| Anti-ASC, IF 1:100 | Novus | NBP1-78-977 |
| Anti-HMGB1, 1: 1000 | Genetex | GTX-101277 |
| Anti-HMGB1, 1: 1000 | abcam | ab18256 |
| Anti- LaminB1, 1: 1000 Blots, 1:250 IF | abcam | AB229025 [EPR22165-121] |
| Anti-H3 citrullinated, 1: 1000 Blots, 1:250 IF | abcam | AB5103 |
| Anti-H3, 1: 1000 | Cell signalling Techonology | 3638S |
| Anti-H4, 1: 1000 | Cell signalling Techonology | 2935S |
| Anti-H2A, 1: 1000 | Cell signalling Techonology | 2578S |
| Anti-H1, 1: 1000 | Abcam | AB134914 [EPR6536] |
| Anti-PAD4, 1: 1000 | Abcam | AB214810 [EPR20706] |
| Anti-NLRC4 1: 1000 | Abcam | ab201792 [EPR19733] |
| Anti-β-actin 1: 1000 | Cell signalling Techonology | 4967S |
| Anti-β-actin 1: 5000 | Sigma-Aldrich | A1978 |
| Goat anti-mouse HRP (1/4000) | SouthernBiotech | 1034-05 |
| Goat-anti-rabbit IgG (H+L), HRP conjugate (1/4000) | Advansta | R-05072-500 |
| Rabbit anti-Goat IgG (H+L) Secondary Antibody, HRP (1/4000) | Invitrogen | 81-1620 |
| Goat anti-rabbit IgG (H&L)Dylight 488 (1/1000) | Immunoreagents | GtxRb-003-D488NHSX |
| Donkey anti-Rabbit IgG Dylight 550 (1/1000) | Immunoreagents | DkxRb-003-D550NHSX |
| Goat anti-mouse IgG Dylight 550 (1/1000) | Immunoreagents | GtxMu-003-D550NHSX |
| Goat anti-mouse IgG Dylight 488 (1/1000) | Immunoreagents | GtxMu-003-D488NHSX |
| Hoechst | Sigma-Aldrich | H6024 |
| WGA-Alexa 633 (1/100) | Thermo Fisher Scientific | W21404 |
| RNA, DNA primers/sequences |  |  |
| MRP8-Cre Fw primers | CGCCGTAAATCAATCGATGAGTTGCTTC |  |
| MRP8-Cre Rv primers | GATGCCGGTGAACGTGCAAAACAGGCTC |  |
| Casp1flox Fw primers | CGAGGGTTGGAGCTCAAGTTGACC |  |
| Casp1flox Rv primers | CACTTTGACTTCTCTAAGGACAG |  |
| Chemicals, peptides and recombinant proteins |  |  |
| DFP (5mM) | Sigma-Aldrich | D0879 |
| GSK484 (10µM) | Cayman Chemical | 17488 |
| ZVAD (20µM) | Invivogen | tlrl-vad |
| Y-VAD (40µM) | Invivogen | inh-yvad |
| Z-DEVD | Selleck | S7312 |
| Z-IETD | Selleck | S7314 |
| Z-LEVD | Abcam | ab120489 |
| SYTOX Green Nucleic Acid Stain (100ng/mL) | Thermo Fisher Scientific | S7020 |
| SYTOX Blue Nucleic Acid Stain (100ng/mL) | Thermo Fisher Scientific | S11348 |
| Glycine | Sigma | G7126 |
| PFA (4%) | Sigma-Aldrich | 1004969011 |
| cOmplete Protease Inhibitor Cocktail | Sigma-Aldrich | 11697498001 |
| Lipofectamine-2000 | Thermo Fisher Scientific | 11668019 |
| Triton X-100 | Sigma-Aldrich | X100-500ML |
| Newborn Calf Serum (FCS) | Thermo Fisher Scientific | 16010159 |
| DMEM | Gibco | 11574486 |
| HEPES | Invitrogen | 15630080 |
| Opti-MEM | Gibco | 11524456 |
| RPMI without phenol red | Gibco | 11564456 |
| HBSS without Calcium, Magnesium or Phenol Red | Gibco | 14-175-095 |
| Clarity Max Western ECL Substrate | Bio-Rad | 1705062 |
| Tris Base Ultrapure | Euromedex | EU1018-A |
| SDS Ultrapure 4X | Euromedex | 1012-A |
| Acrylamide / Bisacrylamide 37.5/1 30% | Euromedex | EU0088-B |
| TEMED | Sigma-Aldrich | T9281 |
| PageRuler Prestained Protein Ladder | Thermo Fisher Scientific | 11822124 |
| Vectashield | Vectorlabs | H-1000-10 |
| MACSxpress Whole Blood Neutrophil Isolation Kit human | Miltenyi Biotech | 130-104-434 |
| Anti-Ly-6G MicroBeads UltraPure, mouse | Miltenyi Biotech | 130-120-337 |
| Critical comercial assays |  |  |
| LDH Cytotoxicity Detection Kit | Takara | MK401 |
| Lactate Dehydrogenase Activity Assay Kit | Sigma | MAK066 |
| IL-1 beta Mouse Uncoated ELISA Kit | Thermo Fisher Scientific | 88-7013-88 |
| IL-1 beta Human ELISA Kit | Invitrogen | KHC0011C |
| Bacterial strains |  |  |
| PP34 | Ina Attrée | [1] |
| PP34^ExoUS142A^ | Ina Attrée | [1] |
| PP34^ΔExoU^ | Ina Attrée | [1] |
| CHA | Ina Attrée | [1] |
| CHA^ΔExoS^ | Ina Attrée | [1] |
| PAO1 | J Buyck | This study |
| PAO1^ΔExoS^ | J Buyck | This study |
| PAO1^ΔExoT^ | J Buyck | This study |
| PAO1^ΔExoY^ | J Buyck | This study |
| PAO1^ΔFliC^ | J Buyck | This study |
| PAO1^ΔExsA^ | J Buyck | This study |
| PAO1^ΔMotABCD^ | J Buyck | This study |
| PAO1^ΔMotABCD/ΔFliC^ | J Buyck | This study |
| Salmonella Typhimurium strain SL1344 | Petr Broz | [2,3] |
| Shigella flexnerii  strain M90T | Petr Broz/Jost Enninga | [2,3] |
| Legionella pneumophilia strain Philadelphia-1 | ATCC | 33152 |
| Burkholderia thailandensis  strain E264 | ATCC | 700388 |
| Listeria monocytogenes  strain EGD | Pascale Cossart | N.A. |
| Burkholderia cenocepacia  strain LMG 16656 | ATCC | BAA-245 |
| Francisella tularensis spp novicida  strain U112 | Petr Broz | [2,3] |
| Escherichia coli  strain K12 | Petr Broz | [2,3] |
| Staphylococcus aureus  strain USA-300 | ATCC | BAA-1556 |
| Vibrio cholera  serovar El tor | Petr Broz | [2,3] |
| Softwares |  |  |
| Prism v.8 |  |  |
| FlowJo v.10 |  |  |
| ImageLab  FiJi |  |  |
| IDEAS software v2.6 (Amnis) |  |  |
| Snapgene |  |  |

**References**

1. Deruelle V, Bouillot S, Job V, Taillebourg E, Fauvarque M-O, Attrée I, et al. The bacterial toxin ExoU requires a host trafficking chaperone for transportation and to induce necrosis. bioRxiv. 2020; 2020.11.04.367706. doi:10.1101/2020.11.04.367706

2. Meunier E, Wallet P, Dreier RF, Costanzo S, Anton L, Rühl S, et al. Guanylate-binding proteins promote activation of the AIM2 inflammasome during infection with Francisella novicida. Nat Immunol. 2015;16: 476–484. doi:10.1038/NI.3119

3. Meunier E, Dick MS, Dreier RF, Schürmann N, Broz DK, Warming S, et al. Caspase-11 activation requires lysis of pathogen-containing vacuoles by IFN-induced GTPases. Nature. 2014;509: 366–370. doi:10.1038/NATURE13157
