# Supplementary figures and images for: Caspase-1-driven neutrophil pyroptosis and its role in host susceptibility to Pseudomonas aeruginosa
Source: PLoS Pathog. 2022 Jul 18;18(7):e1010305. doi: 10.1371/journal.ppat.1010305 (PMC9345480; doi:10.1371/journal.ppat.1010305)

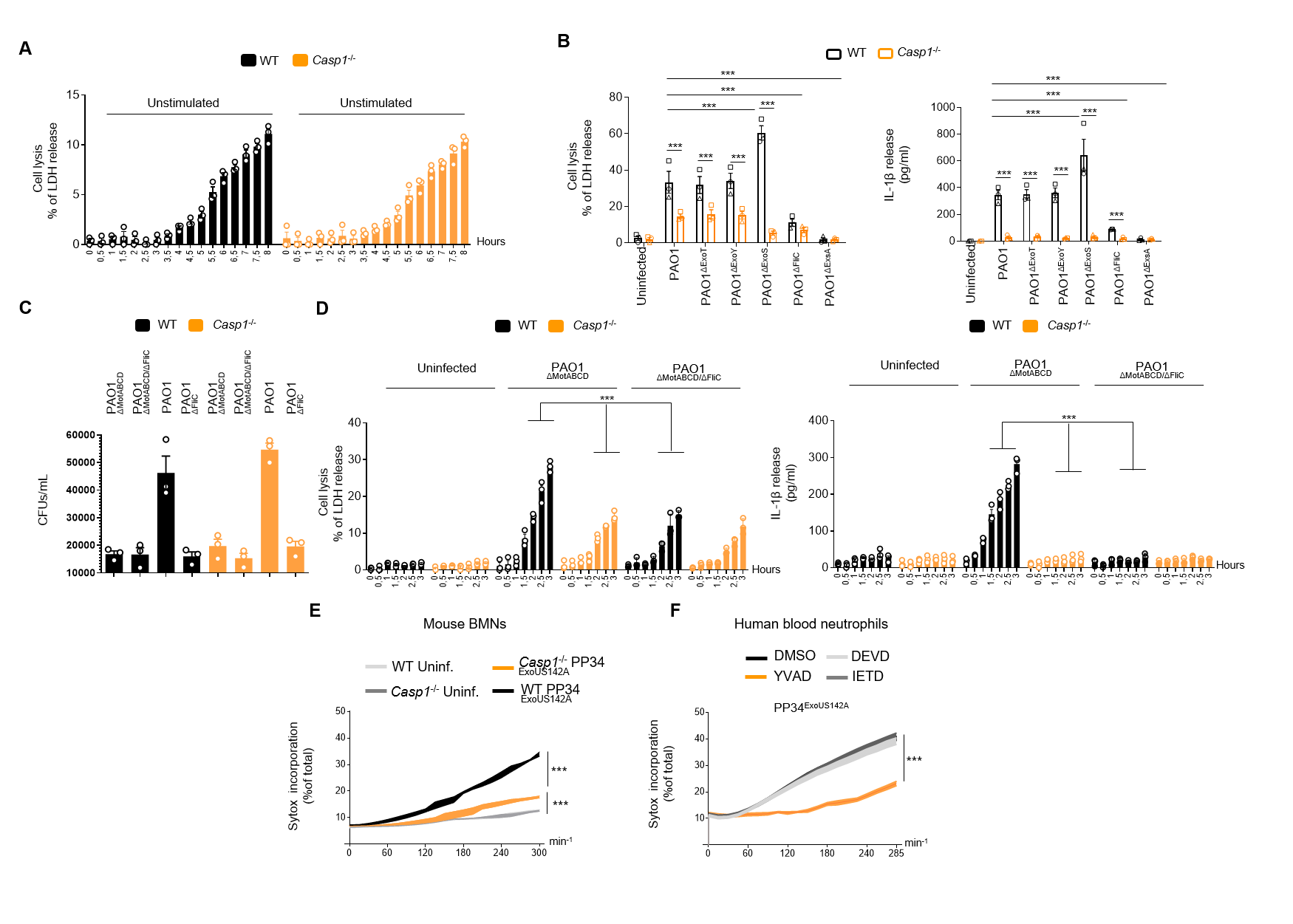

Supplement: S1 Fig — A. Measure of basal cell lysis (release of LDH) in WT and Casp1-/- murine Bone Marrow Neutrophils (BMNs) in culture for the indicated times. Values are expressed as mean ± SEM. Graphs show combined values from three independent experiments. B. Measure of cell lysis (release of LDH) and IL-1β release in WT or Casp1-/- murine Bone Marrow Neutrophils (BMNs) infected for 3 hours with Pseudomonas aeruginosa PAO1 and its isogenic mutants lacking T3SS expression (PAO1ΔExsA), Flagellin (PAO1ΔFliC) or T3SS-derived toxins ExoS, ExoY, ExoT (PAO1ΔExoS, PAO1ΔExoY, PAO1ΔExoT) at a multiplicity of infection (MOI) of 10. ***p ≤ 0.001, Two-Way Anova with multiple comparisons. Values are expressed as mean ± SEM. Graphs show combined values from three independent experiments. Values are expressed as mean ± SEM. C. Measure of bacterial uptake (Colony-forming Units, CFUs) in WT or Casp1-/- BMNs infected for 45 minutes with Pseudomonas aeruginosa PAO1 and its isogenic mutants lacking Flagellin (PAO1ΔFliC), Flagellin motors MotABCD (PAO1ΔMotABCD) or both Flagellin and Flagellin motors MotABCD (PAO1ΔFliC/ΔMotABCD) at a MOI of 10. Here, due to their lack of motility, bacteria were centrifuged for 5 min/1000 rpm to ensure neutrophil/bacterial contact. ***p ≤ 0.001, Two-Way Anova with multiple comparisons. Values are expressed as mean ± SEM. Graph show one experiment representative of three independent experiments. D. Measure of cell lysis (release of LDH) and IL-1β release in WT and Casp1-/- murine Bone Marrow Neutrophils (BMNs) infected for the indicated times with PAO1ΔMotABCD or PAO1ΔFliC/ΔMotABCD at an MOI of 10. Here, due to their lack of motility, bacteria were centrifuged for 5 min/1000 rpm to ensure neutrophil/bacterial contact. ***p ≤ 0.001, Two-Way Anova with multiple comparisons. Values are expressed as mean ± SEM. Graphs show combined values from three independent experiments. E. Measure of the percentage of cells with plasma membrane permeabilization over time using SYTOX Green [file ppat.1010305.s007.tif]

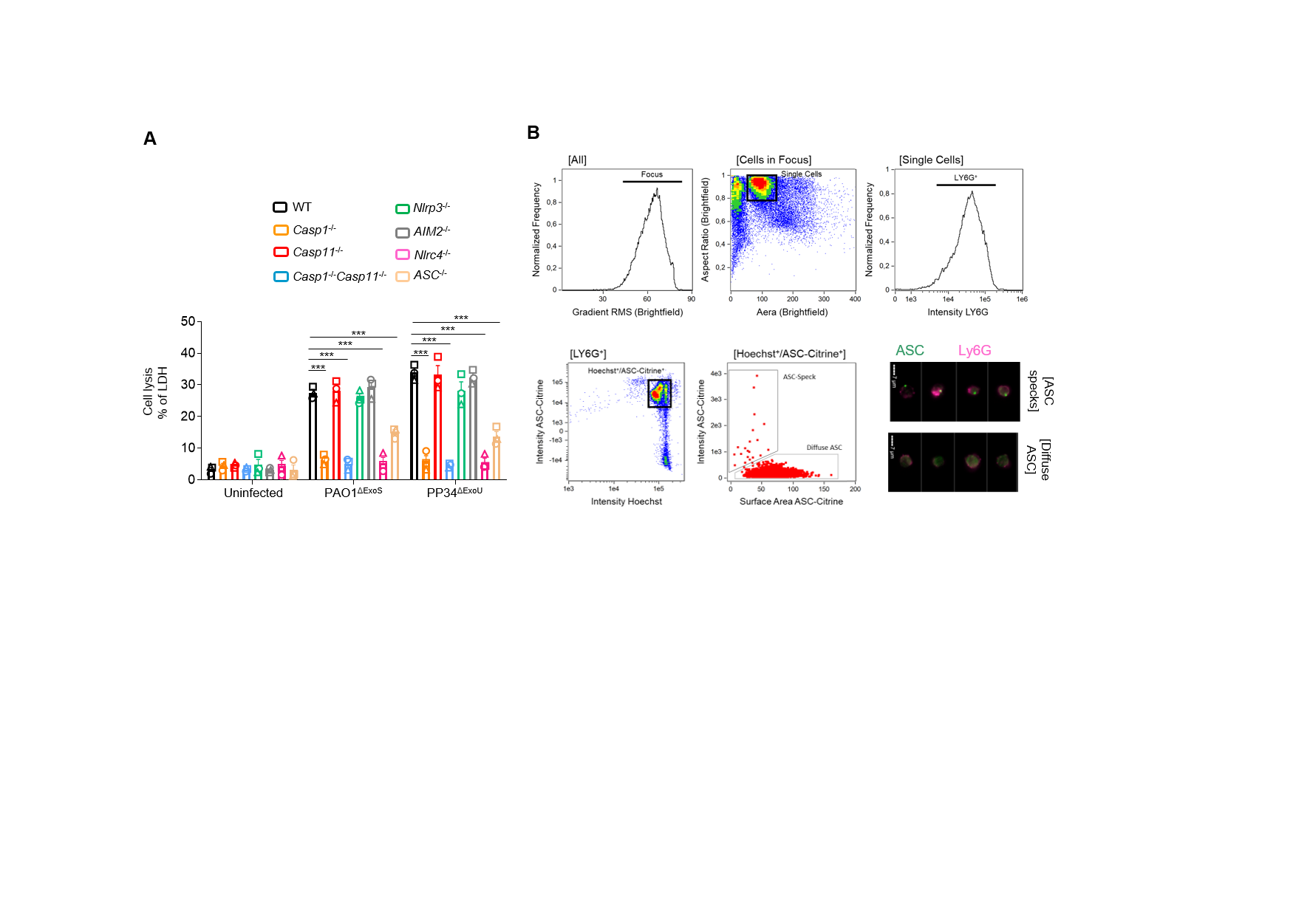

Supplement: S2 Fig — A. Measure of cell lysis (release of LDH) in WT, Casp1-/-, Casp1-/-, Casp11-/-, Casp1-/-Casp11-/-, Nlrp3-/-, AIM2-/—, Nlrc4-/- and ASC-/- murine Bone Marrow Neutrophils (BMNs) infected for 3 hours with Pseudomonas aeruginosa pyroptotic strains PP34ExoUS142A (MOI 2) and PAO1ΔExoS (MOI 10). ***p ≤ 0.001, Two-Way Anova with multiple comparisons. Values are expressed as mean ± SEM. Graphs show combined values from three independent experiments. B. Gating strategy used to evaluate inflammasome activation in neutrophils was performed as follow: (i) a gate was set on cells in focus [Cells in Focus] and (ii) a sub-gate was created on single cells [Single Cells]. Then we gated first on (iii) LY6G+ Neutrophils [LY6G+] and second on (iv) ASC-citrine+ and Hoechst+ cells [Hoechst+/ASC-Citrine+] within LY6G+ population. (v) To distinguish cells with active (ASC-speck) versus inactive inflammasome (Diffuse ASC), we plotted the Intensity with the area of ASC-citrine. This strategy allow to distinguish cells with active inflammasome that were visualized and quantified. (TIF) [file ppat.1010305.s008.tif]

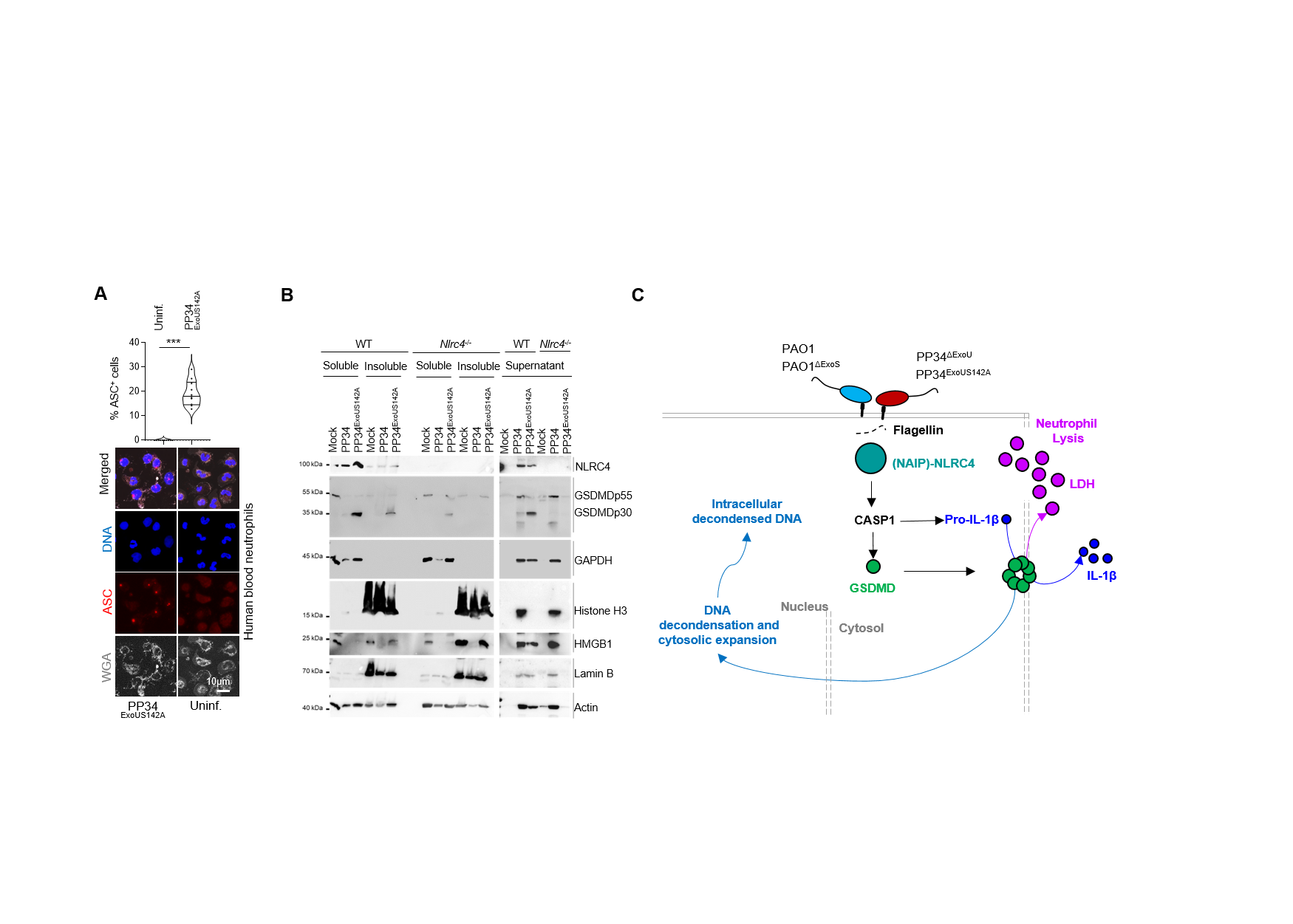

Supplement: S3 Fig — A. Confocal microscopy observations and quantification of primary human blood neutrophils infected for 3 hours with P. aeruginosa pyroptotic strain PP34ExoUS142A (MOI 2) and harboring ASC complexes, decondensed DNA and plasma membrane. Nucleus (blue) was stained with Hoechst; ASC is in Red (anti-ASC); Plasma membrane is in grey (WGA). Scale bar 10μm. Images are representative of one experiment performed three times with at least 6–10 fields neutrophils observed/ quantified for ASC specks ratios. ***p ≤ 0.001, T-test with Bonferroni correction. B. Immunoblotting observation of Histone 3, HMGB1, Lamin B1, GAPDH, Actin, Gasdermin D (GSDMD) and NLRC4 in cellular soluble and insoluble fractions as well as in the supernatant from WT and Nlrc4-/- murine BMNs infected with P. aeruginosa NETotic strain PP34 or pyroptotic strain PP34ExoUS142A (MOI 2) for 3 hours. Immunoblots show one experiment performed two times. C. Overview of the different steps induced in neutrophils during NLRC4 inflammasome activation by various Pseudomonas aeruginosa strains. (TIF) [file ppat.1010305.s009.tif]

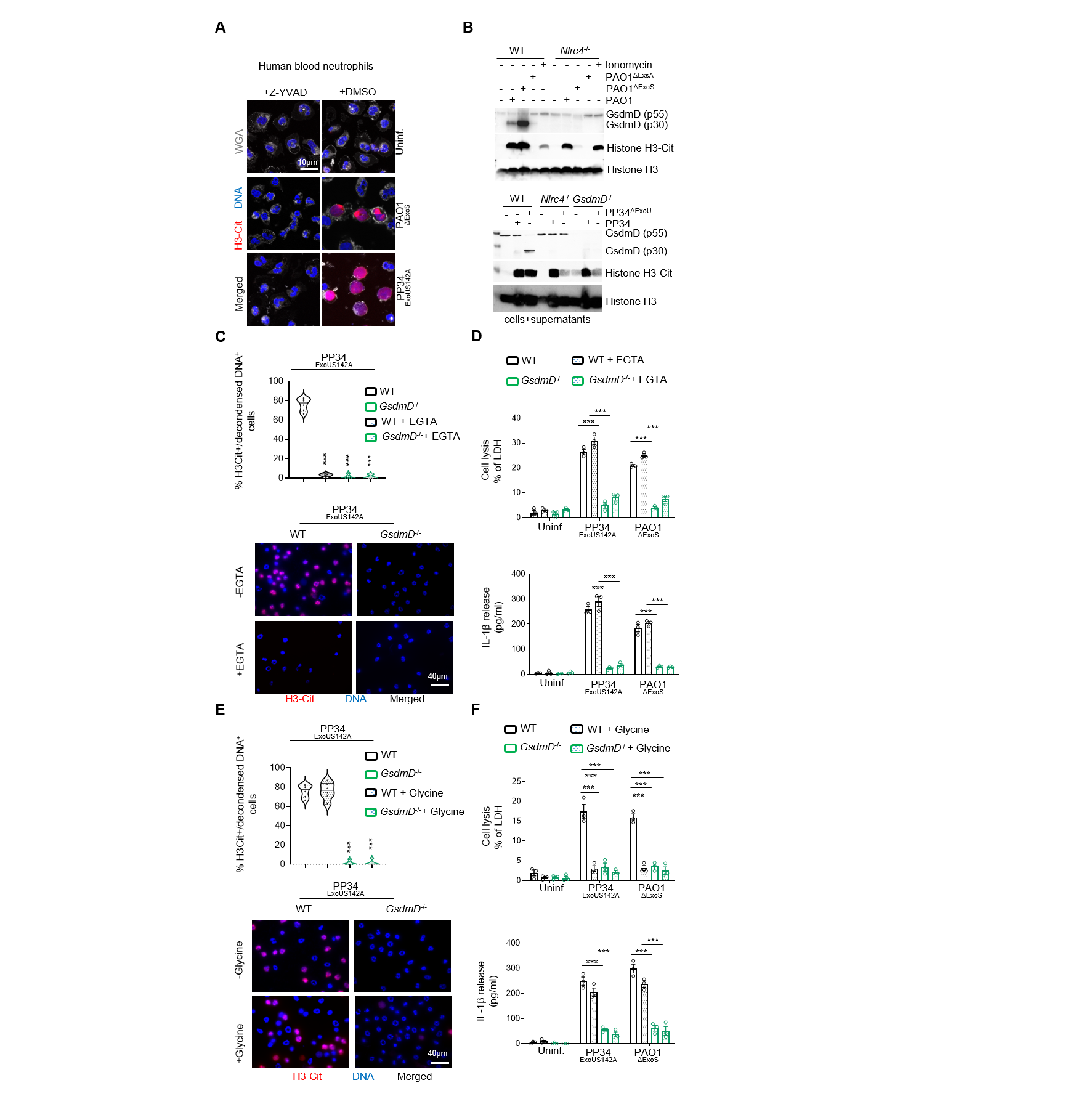

Supplement: S4 Fig — A. Confocal microscopy observations and quantifications of human blood neutrophils infected for 3 hours with PAO1ΔExoS (MOI 10) or PP34ExoUS142A (MOI 2) in presence/absence of Caspase-1 inhibitor Z-YVAD (20μM) and harboring Citrullinated Histone 3 (H3-Cit), decondensed DNA and nuclear membrane (LaminB1). Nucleus (blue) was stained with Hoechst; Citrullinated Histone-3 is in red (anti H3-Cit); plasma membrane is in grey (WGA staining). Scale bar 10μm. Images are representative of one experiment performed three times with at least 150 neutrophils observed/ experiment. B. Immunoblotting of Citrullinated Histone 3 (H3Cit), total Histone 3 and preformed and cleaved Gasdermin-D (p55/p30) in WT, Nlrc4-/- and GsdmD-/- BMNs treated with Ionomycin (10μM, 3 hours) or infected for 3 hours with PAO1, PAO1ΔExoS, PAO1ExsA- (MOI 10) or with PP34, PP34ΔExoU (MOI 2). Immunoblots show combined lysates and supernatants from one experiment performed three times. C. Confocal microscopy observations and quantifications of WT and GsdmD-/- BMNs infected for 3 hours with P. aeruginosa pyroptotic strain PAO1ExoUS142A (MOI 2) in presence/absence of EGTA (10mM) and harboring Citrullinated Histone 3 (H3-Cit) and decondensed DNA. Nucleus (blue) was stained with Hoechst; Histone-3 Citrullination is in red (Anti-H3Cit staining). For quantifications, the percentage of cells positives for H3Cit (H3-Cit+) and decondensed DNA was determined by quantifying the ratios of cells positives for H3Cit and decondensed DNA over the total amount of cells. At least 6 fields from n = 3 independent experiments were analyzed. Values are expressed as mean ± SEM. ***p ≤ 0.001, Two-Way Anova with multiple comparisons. D. Measure of cell lysis (release of LDH) and IL-1β release in WT or GsdmD-/- BMNs infected for 3 hours with PP34ExoUS142A (MOI2) or PAO1ΔExoS (MOI 10) in presence/absence of EGTA (10mM). ***p ≤ 0.001, Two-Way Anova with multiple comparisons. Values are expressed as mean ± SEM. Graphs show combined values [file ppat.1010305.s010.tif]
