# Supplementary material for: Caspase-1-driven neutrophil pyroptosis and its role in host susceptibility to Pseudomonas aeruginosa
Source: PLoS Pathog. 2022 Jul 18;18(7):e1010305. doi: 10.1371/journal.ppat.1010305 (PMC9345480; doi:10.1371/journal.ppat.1010305)
Supplement: Attachment — Submitted filename: rebuttal.docx [file ppat.1010305.s011.docx]

Dear Dr. Meunier,

Thank you very much for submitting your manuscript "Caspase-1-driven neutrophil pyroptosis promotes an incomplete NETosis upon Pseudomonas aeruginosa infection" for consideration at PLOS Pathogens. As with all papers reviewed by the journal, your manuscript was reviewed by members of the editorial board and by several independent reviewers. In light of the reviews (below this email), we would like to invite the resubmission of a significantly-revised version that takes into account the reviewers' comments.

The reviewers have identified experimental important issues that should be addressed. In addition, the text as written have issues with clarity. Examples include:

1. Description of results (example is Fig. 1B and Fig 2A-E). So brief that it doesn't seem to help the readers understand the data presented. Expanding these and other sections would be helpful.

2. Reference to Fig. 3E on line 265 seems incorrect.

3. Legends for some figure panels and figures can be improved for clarity. Fig 1D - are these showing merged micrographs or separate brightfield and SYTOX green images. It is not clear.

4. Overall, the authors are encouraged to have the language reviewed for clarity. A reduction in the use of quotation marks could be helpful.

We cannot make any decision about publication until we have seen the revised manuscript and your response to the reviewers' comments. Your revised manuscript is also likely to be sent to reviewers for further evaluation.

When you are ready to resubmit, please upload the following:

[1] A letter containing a detailed list of your responses to the review comments and a description of the changes you have made in the manuscript. Please note while forming your response, if your article is accepted, you may have the opportunity to make the peer review history publicly available. The record will include editor decision letters (with reviews) and your responses to reviewer comments. If eligible, we will contact you to opt in or out.

[2] Two versions of the revised manuscript: one with either highlights or tracked changes denoting where the text has been changed; the other a clean version (uploaded as the manuscript file).

Important additional instructions are given below your reviewer comments.

Please prepare and submit your revised manuscript within 60 days. If you anticipate any delay, please let us know the expected resubmission date by replying to this email. Please note that revised manuscripts received after the 60-day due date may require evaluation and peer review similar to newly submitted manuscripts.

Thank you again for your submission. We hope that our editorial process has been constructive so far, and we welcome your feedback at any time. Please don't hesitate to contact us if you have any questions or comments.

***********************

The reviewers have identified experimental important issues that should be addressed. In addition, the text as written have issues with clarity. Examples include:

1. Description of results (example is Fig. 1B and Fig 2A-E). So brief that it doesn't seem to help the readers understand the data presented. Expanding these and other sections would be helpful.

2. Reference to Fig. 3E on line 265 seems incorrect.

3. Legends for some figure panels and figures can be improved for clarity. Fig 1D - are these showing merged micrographs or separate brightfield and SYTOX green images. It is not clear.

4. Overall, the authors are encouraged to have the language reviewed for clarity. A reduction in the use of quotation marks could be helpful.

We thank our editor for all those helpful suggestions. They grandly improved the relevance, clarity and rigor of the work presented in this study.

All mentioned aspects have been addressed in detail (experimentally and by formatting) in the rebuttal (below) and all along the revised manuscript.

Reviewer's Responses to Questions

Part I - Summary

Please use this section to discuss strengths/weaknesses of study, novelty/significance, general execution and scholarship.

**Reviewer #1:** In the present manuscript, Santoni et al describe a phenomenon of incomplete netosis triggered by inflammasome activation by certain Pseudomonas strains in neutrophils. This is interesting as neutrophils have been believed to be largely refractory to pyroptosis upon bacterial infection under normal circumstances. The authors attribute some of the observed accompanying phenomena such as histone citrunillation to PAD4 activation but fail to uncover the reason for P. aeruginosa specific induction of neutrophil pyroptosis over other bacteria. They demonstrate a role for Caspase 1 in neutrophils with a neutrophil specific Casp1-Cre mouse line in the infectivity levels of Pseudomonas in vivo, extending on previously published data with whole Caspase-1 knockouts. Their observations suggest that NETosis is a very controlled process with several unknown aspects remaining.

Several of the observations require additional quantifications to allow the conclusions drawn, and the manuscript should be carefully edited for grammar and correct referencing of figures and panels.

We thank our reviewer for the very helpful suggestions. Overall, they strongly improve the clarity of our study.

**Reviewer #2:** Inflammasomes are multiprotein complexes that activate caspase-1 and -11. In most cases, activation of these inflammatory caspases promote IL-1b maturation and a lytic form of cell death known as pyroptosis. However, in neutrophils, caspase-1 activation appears to resist pyroptosis through a poorly understood mechanism. Here, Santoni and co-workers show that neutrophils are not universally resistant to caspase-1-dependent pyroptosis as previously reported. They found that various strains of Pseudomonas aeruginosa can in fact trigger caspase-1-dependent pyroptosis in neutrophils, and some of these strains promote nuclear membrane rupture and histone citrullination in the absence of cell lysis, which the authors termed ‘incomplete NETosis’. Overall, this is an interesting manuscript and will be of interest to the microbiology and innate immunity community. However, this is a complicated study, and the presentation of the manuscript can be improved significantly. The labelling and definition require a lot of work – while the introduction, abstract and summary are quite well written, the results are very hard to follow, due to typo mistakes and inconsistent labelling. For example (also mentioned in point 5, 9), does PP34 normally express ExoU? If so, it is redundant to write PP34ExoU, since that implies exoU is introduced into the strain. Are the authors referring to the same strain when they mention ExoU- and ExoUS142A? Note that these are not the same. I suggest the to use the standard nomenclature for describing bacterial strains - (delta Greek sign)ExoU for knockout and not ExoU-, and clearly differentiate when using a catalytic inactive versus a knockout. There are also other experimental controls and concerns that should be addressed.

We thank our reviewer for his/her insightful comments. We have tried addressing them experimentally or by editing our manuscript. We hope, they will convince our reviewer of the scientific relevance of our study.

Part II – Major Issues: Key Experiments Required for Acceptance

Please use this section to detail the key new experiments or modifications of existing experiments that should be absolutely required to validate study conclusions.

Generally, there should be no more than 3 such required experiments or major modifications for a "Major Revision" recommendation. If more than 3 experiments are necessary to validate the study conclusions, then you are encouraged to recommend "Reject".

**Reviewer #1:**

- For the Pseudomonas strain comparison experiments shown in figure 1C and E and S1, it is important to verify that the strains are equally infectious to be able to draw any conclusion on the role of effectors. Also, aflagellated bacteria are expected to be less infectious due to their limited motility. It is not clear from the methods whether the authors i.e. spun Fla-negative bacteria onto cells to help with infection. Ideally, the authors would plate for numbers of intracellular bacteria at an early, pre-death timepoint or something similar

We fully agree. Accordingly, we have performed experiments with *P. aeruginosa* strain PAO1 in which the Flagellin motors have been genetically removed (MotABCD) (doi: 10.1128/IAI.00054-13, doi: 10.1038/s41420-018-0068-z). The generated strains (PAO1^ΔMotABCD^ and PAO1^ΔMotABCD/ΔFliC^) are equally phagocytosed by neutrophils and are known to trigger NLRC4 inflammasome activation in macrophages as they still express non motile flagellin (doi: 10.1128/IAI.00054-13, doi: 10.1038/s41420-018-0068-z). Here, we could show that PAO1^ΔMotABCD^ but not PAO1^ΔMotABCD/ΔFliC^ triggered a Caspase-1-dependent neutrophil pyroptosis and IL-1beta release over time.

As P. aeruginosa strains lacking Flagellin expression or motility, are less efficiently recognized/phagocytosed by immune cells, we did centrifuge both PAO1^ΔMotABCD^ and PAO1^ΔMotABCD/ΔFliC^ on neutrophils to force bacteria-neutrophil contact **(Supplemental Figure 1, below and in manuscript).**

Hence, we are confident that Flagellin plays a major role on the activation of Caspase-1-depndent pyroptosis of neutrophils upon *P. aeruginosa* infection. Those results have been included in Supplemental Figure 1 of the revised manuscript.

**From Supplemental Figure 1. Multiple *P. aeruginosa* strains triggers Caspase-1-dependent pyroptosis in neutrophils. (S1C, S1D in the manuscript)**

Measure of bacterial uptake (Colony-forming Units, CFUs) in WT or *Casp1*^-/-^ BMNs infected for 45 minutes with *Pseudomonas aeruginosa* PAO1 and its isogenic mutants lacking Flagellin (PAO1^ΔFliC^), Flagellin motors MotABCD (PAO1^ΔMotABCD^) or both Flagellin and Flagellin motors MotABCD (PAO1^ΔFliC/ΔMotABCD^) at a MOI of 10. Here, due to their lack of motility, bacteria were centrifuged for 5 min/1000 rpm to ensure neutrophil/bacterial contact. ***p ≤ 0.001, Two-Way Anova with multiple comparisons. Values are expressed as mean ± SEM. Graph show one experiment representative of three independent experiments.

Measure of cell lysis (release of LDH) and IL-1β release in WT and *Casp1*^-/-^ murine Bone Marrow Neutrophils (BMNs) infected for the indicated times with PAO1^ΔMotABCD^ or PAO1^ΔFliC/ΔMotABCD^ at an MOI of 10. Here, due to their lack of motility, bacteria were centrifuged for 5 min/1000 rpm to ensure neutrophil/bacterial contact. ***p ≤ 0.001, Two-Way Anova with multiple comparisons. Values are expressed as mean ± SEM. Graphs show combined values from three independent experiments.

-Imaging in 3A, 3B and 3C needs to be quantified over multiple images. It is also not clear why the magnification for uninfected cells in 3A is different than for infected cells

Accordingly, we now provide image quantifications for the Figure 3, included in the revised manuscript.

Indeed, magnification of the controls was different, this has now been homogenized in order to avoid discrepancies between image scales.

**From Figure 3. Inflammasome signaling in neutrophils promotes both pyroptotis and DNA decondensation but not extracellular DNA expulsion**

**A.** Scanning electron microscopy (SEM) observations and quantifications of pyroptosis in WT, *Casp1*^-/-^ and *GsdmD*^-/-^ BMNs 3 hours after infection with *P. aeruginosa* strains PAO1, PAO1^ΔExoS^, PP34 and PP34^ExoUS142A^ at an MOI of 10 (PAO1 strains) and 2 (PP34 strains). Images are representative of one experiment performed 3 times. Scale bars are directly indicated in the Figure. For quantifications, the percentage of cells exhibiting extracellular DNA was determined by quantifying the ratios of cells positives for extracellular DNA over the total of cells. 6-10 fields from n=3 independent experiments were analyzed. Values are expressed as mean ± SEM. ***p ≤ 0.001, Two-Way Anova with multiple comparisons.

**B.** Confocal microscopy observations and quantifications of WT, *Casp1*^-/-^ and *GsdmD*^-/-^ BMNs infected for 3 hours with the pyroptotic strain PP34^ExoUS142A^ (MOI 2) and harboring ASC complexes, decondensed DNA and nuclear membrane (LaminB1). Nucleus (blue) was stained with Hoechst; LaminB1 is in red (anti LaminB1); ASC is in Green (anti-ASC); plasma membrane is in grey (WGA staining). Scale bar 10µm. Images are representative of one experiment performed three times. For quantifications, the percentage of cells with ASC complexes and nuclear DNA was determined by quantifying the ratios of cells positives for ASC speckles and nuclear DNA. 6-10 fields from n=3 independent experiments were analyzed. Values are expressed as mean ± SEM. ***p ≤ 0.001, Two-Way Anova with multiple comparisons.

**C.** Representative time lapse fluorescence microscopy images and quantifications of ASC-Citrine murine BMNs infected with the NETotic strain PP34 or the pyroptotic strain PP34^ExoUS142A^ (MOI 2) for 9 hours (540 minutes). Nucleus (blue) was stained with Hoechst; ASC is in yellow (ASC-Citrine); plasma membrane is in green (WGA staining); plasma membrane permeabilization is stained in red (cell impermanent DNA dye Propidium Iodide, PI). Images are representative of one movie out of three independent movies. For quantifications, the percentage of cells harboring decondensed DNA and/or extracellular decondensed DNA was determined by quantifying the ratios of cells with decondensed DNA (area surface) and of cells with decondensed DNA crossing WGA staining (plasma membrane). At least 6 fields containing each 20-30 cells were quantified. Scale bar 10µm. ***p ≤ 0.001, T-test with Bonferroni correction.

-While GSK484 seems to be a very specific inhibitor, it would be nice to see the H3 citrullin staining and quantification as in 3E in Pad4 knockouts as used in the supplemental data.

As suggested by our reviewer, we have included a new set of experiments using WT and Pad4-/- murine neutrophils infected with the pyroptotic strain of P. aeruginosa PP34S142A. Contrary to WT neutorphils, Pad4-/- neutrophils exhibit a strong defect at citrullinating Histones but also at promoting DNA decondensation. These experiments have been included in the new Figure 4 in our revised manuscript.

**From Figure 4. Canonical inflammasome activation-induced DNA decondensation requires PAD4-dependentent Histone Citrullination and occurs independently of cell shrinkage (Fig. 4E).**

Confocal microscopy observations and quantifications of WT and Pad4-/- BMNs infected for 3 hours with P. aeruginosa pyroptotic strain PAO1^ΔExoS^ (MOI 10) and harboring ASC complexes, Citrullinated Histone 3 (H3-Cit), decondensed DNA and plasma membrane (WGA). Nucleus (blue) was stained with Hoechst; Histone-3 Citrullination is in red (Anti-H3Cit staining); plasma membrane is in green (WGA staining), ASC is in grey (Anti-ASC). Scale bar 10µm. For quantifications, the percentage of cells with ASC complexes, nuclear DNA or positives for H3Cit (H3-Cit+) was determined by quantifying the ratios of cells positives for ASC speckles, nuclear DNA or H3Cit. At least 10 fields from n=3 independent experiments were analyzed. Values are expressed as mean ± SEM. ***p ≤ 0.001, Two-Way Anova with multiple comparisons.

**Reviewer #2:** Major points

1. Neutrophils are extremely short-lived and sensitive cells and should be undergoing 20-30% basal cell death at the end of inflammasome assays. Why are the values for LDH release for untreated neutrophils 0? I believe that is not possible. Authors should also indicate untreated values for all other experiments to determine the baseline of neutrophil cell death.

We acknowledge our reviewer comment. Here, we used positive selection for murine neutrophil isolation and purification and negative selection for human neutrophil selection/isolation. There was no priming or pre-activation step before infections. In our settings, we infect neutrophils for 3 hours. Indeed, we performed kinetics of neutrophil lysis over time to frame the window in which neutrophil lysis might interfere with *P. aeruginosa*-induced Caspase-1-dependent pyroptosis. Here, we observed few LDH release 3 hours post neutrophils isolation and purification. It mostly started after 4-4.5hours of culture. In these experimental settings, we explain this by the fact that neutrophils that spontaneously release some LDH are apoptotic neutrophils (https://www.ncbi.nlm.nih.gov/pmc/articles/PMC4979515/). Hence, there is a delay between neutrophil apoptosis and LDH release from the permeabilized blebs (https://pubmed.ncbi.nlm.nih.gov/23852598/) (https://www.nature.com/articles/ncomms14128). Accordingly, Chen and colleagues, in their study on the role of Gasdermin E to promote post-apoptosis LDH release measured LDH levels in supernatants after 6 hours of culture whereas we do measure LDH in our experiments only after 3 hours at best (https://doi.org/10.1073/pnas.2101189118). In addition, Sytox incorporation allows measuring plasma membrane permeabilization, a process that, during apoptosis, occurs only after blebs formation, and just before the appearance of LDH in the supernatant. Accordingly, the kinetics performed by Chen et al (as well as our kinetics) show that spontaneous Sytox incorporation in resting neutrophils increases after 3.5-4 hours of culture, a time that is just before the measure of increased levels of LDH release in neutrophil cultures. Hence, we strongly believe that the low LDH levels, and sometimes the absence of robust LDH signal in our experimental controls, are mostly due to the time point we use for LDH measures as described in the following figures.

**We also included all values for LDH in untreated neutrophils for all experiments in the revised version of the manuscript.**

**Kinetics of LDH release between resting Wt and Casp1-/- neutrophils have now been included in Supplemental Fig 1. Of the revised manuscript.**

A. Measure of basal cell lysis (release of LDH) in WT and *Casp1*^-/-^ murine Bone Marrow Neutrophils (BMNs) in culture for the indicated times. Values are expressed as mean ± SEM. Graphs show combined values from three independent experiments.

2. Early studies from different labs have shown that bacterial infection triggers at best 30-40% LDH release in neutrophils, a finding that the authors reproduced in Figure S2C too. Why are the values so high in Figure 1? Can the authors explain the discrepancy with the field? Are the neutrophils activated during purification?

We do not preactivate neutrophils. We agree that some microbial species induce between 30-40% cell lysis of neutrophils, a process we also observe with *P. aeruginosa* pyroptotic strains. However, studies on *S. flexneri* (<https://www.ncbi.nlm.nih.gov/pmc/articles/PMC97281/>) or *S. aureus* (<https://ashpublications.org/blood/article/129/24/3237/36314/Lysis-of-human-neutrophils-by-community-associated> and <https://www.karger.com/Article/Pdf/317134>) have also found extremely high levels of neutrophil lysis upon infection, an observation we also made.

Here, we think that the type of bacteria used, the MOI but also the microbial fitness after cultures are important parameters to keep in mind for those observations of hyper neutrophil lysis.

3. Figure 1B: Author mentioned that Salmonella infection triggers caspase-1-dependent pyroptosis in human neutrophils, but the Figure indicates ‘CHA’ but not Salmonella. What is CHA?

We apologize, this was a remnant from a previous version with a figure containing neutrophils infected with Salmonella. This has been modified. CHA stands for another *P. aeruginosa* strain (<https://doi.org/10.1093/infdis/jiw295>).

4. Y-VAD blocks both caspase-1 and -4. How can the authors be sure that the effect of Y-VAD is not due to blockade of caspase-4?

Indeed, at certain concentrations YVAD can also exhibit some Caspase-4 inhibitory action. Here, we also used a Caspase-4/5 inhibitor, namely Z-LEVD, that did not show inhibitory action on human neutrophils infected with P. aeruginosa strains PAO1 and CHA, although YVAD efficiently decreased P. aeruginosa-induced pyroptosis and IL1beta release. Hence, we are confidents that in human neutrophils Caspase-1 also contributes to neutrophil pyroptosis upon P. aeruginosa infection. These results have been included in the revised version of the manuscript (Figure 1)

**From Figure 1.** Measure of cell lysis (release of LDH) and IL-1β release in human blood neutrophils infected for 3 hours with Pseudomonas aeruginosa strains PAO1 or CHA (MOI 5) in presence/absence of various Caspase inhibitors, Z-VAD (pan Caspase, 20µM), Z-YVAD (Casp1 inhibitor, 40µM), Z-DEVD (Casp3 inhibitor, 40µM), Z-IETD (Casp8 inhibitor, 40µM) or Z-LEVD (Casp4/5 inhibitor, 40µM). **p ≤ 0.01 ***p ≤ 0.001, Two-Way Anova with multiple comparisons. Values are expressed as mean ± SEM. Graphs show values from three independent experiments with one different donor each time.

5. The section on PP34ExoU is very confusing and needs to be re-written (lines 237-246). Am I right to say that ExoU-deficient strains also trigger caspase-1-dependent pyroptosis in neutrophils?

We apologize for the lack of clarity. Hence, for more clarity, we have included all along the figures and the text, further precisions on the type of *P. aeruginosa* strains that trigger Caspase-1-dependent neutrophil pyroptosis.

In addition, we also rewrote the sections about ExoU strains, in order to help the reading.

“*P. aeruginosa* strains PAO1 and CHA belong to the 70% of the *P. aeruginosa* strains that express the Exotoxin S (ExoS). As 30% of *P. aeruginosa* strains do not express the Exotoxin S (ExoS) but instead are characterized as expressing the extremely lytic phospholipase toxin ExoU, we expanded our analysis to neutrophils infected with a strain expressing a catalytically inactive mutant of ExoU (PP34^ExoUS142A^). In addition, we examined the response of neutrophils to a *P. aeruginosa* strain that naturally expresses ExoU (PP34 strain) or a mutant thereof that is genetically deficient for ExoU (PP34^ΔExoU^). These experiments showed that the *P. aeruginosa* PP34 strain potently induces lysis of *Casp1*^-/-^ or *GsdmD*^-/-^ neutrophils (**Fig. 1F**, **S1E Fig.**). However, both human and murine neutrophils infected with PP34^ΔExoU^ or PP34^ExoUS142A^ showed robust Caspase-1-dependent neutrophil lysis, plasma membrane permeabilization and IL-1β release (**Fig. 1F**, **S1E, F Fig.**). These results suggest that Caspase-1 and GSDMD-dependent pyroptosis is masked or suppressed by ExoU activity in the *P. aeruginosa* PP34 strain. ”

6. PP34ExoU/FliC- is unable to trigger ASC speck in vivo but the lack of inflammasome activation for the double mutant has not been validated in vitro. Does this mean flagellin is required for NLRC4 activation but ExoU suppresses it? Why is that so?

Accordingly, we validated the double KO strain in vitro (figure below).

**Refer to Figure 2D.** Measure of cell lysis (release of LDH) in WT and Nlrc4-/- BMNs infected for 3 hours with P. aeruginosa mutant strains PP34ΔExoU or PP34ΔExoU/ΔFliC at a multiplicity of infection (MOI) of 2 and Imagestream experiments and quantifications of in vivo formation of ASC specks in bronchoalveolar (BALs) neutrophils from ASC-Citrine mice intranasally infected with 1.105 PP34ΔExoU or PP34ΔExoU/ΔFliC for 6 hours..

The gating strategy used to evaluate inflammasome activation in neutrophils was performed as follow: (i) a gate was set on cells in focus [Cells in Focus] and (ii) a sub-gate was created on single cells [Single Cells]. Then we gated first on (iii) LY6G+ Neutrophils [LY6G+] and second on (iv) ASC-citrine+ and Hoechst+ cells [Hoechst+/ASC-Citrine+] within LY6G+ population. (v) To distinguish cells with active (ASC-speck) versus inactive inflammasome (Diffuse ASC), we plotted the Intensity with the area of ASC-citrine. This strategy allows to distinguish cells with active inflammasome that were visualized and quantified.

For Imagestream experiments ***p ≤ 0.001, T-test with Bonferroni correction. Values are expressed as mean ± SEM. Graphs show one experiment representative of two independent experiments.

For neutrophil in vitro experiments, ***p ≤ 0.001, Two-Way Anova with multiple comparisons. Values are expressed as mean ± SEM. Graphs show combined values from three independent experiments.

Regarding the putative role of ExoU at inhibiting inflammasome activation by Flagellin, this is currently investigated by different groups. Our finding mostly suggest that ExoU lyses neutrophils extremely quickly and that this might also explain the lack of pyroptosis phenotype with ExoU-expressing P. aeruginosa strains (Figures A and B below). Here, we used PP34 (P. aeruginosa that naturally expresses ExoU), MAFP, an inhibitor of phospholipase activity (inhibitor of ExoU activity) or strains that lack ExoU activity (PP34^ExoUS142A^) and measured over time the ability of neutrophils to exhibit plasma membrane permeabilization (SYTOX incorporation). As clearly observed in figures, ExoU-expressing *P. aeruginosa* (PP34) induced faster plasma membrane permeabilization than PP34^ExoUS142A^ or MAFP-treated neutrophils. This suggests to us that the extremely fast lysis of neutrophils induced by ExoU might be responsible of the lack of obvious Caspase-1-dependent pyroptosis in response to ExoU-expressing strains.

Measure of plasma membrane permeabilization over time using SYTOX Green incorporation in murine (A) BMNs or in human blood neutrophils (B) infected with PP34ExoU and its isogenic catalytically inactive mutant PP34ExoUS142A at a multiplicity of infection (MOI) of 2 in presence/absence of the phospholipase inhibitor MAFP (20µM). ***p ≤ 0.001, T-test with Bonferroni correction. Values are expressed as mean ± SEM. Graphs show one experiment representative of three independent experiments.

Finally, measure of cell lysis (LDH release) showed that strains lacking ExoU expression (PP34deltaExoU) triggered Caspase-1-depndent neutrophil lysis, but not PP34 strain, naturally expressing ExoU.

**Refer to Figure 1F**. Measure of cell lysis (release of LDH) in WT, *Casp1*^-/-^, *Casp11*^-/-^, *Casp1*^-/-^*Casp11*^-/-^ and *GsdmD*^-/-^ murine Bone Marrow Neutrophils (BMNs) infected for 3 hours with *Pseudomonas aeruginosa* PP34 strain or its isogenic mutant PP34^ΔExoU^ at a multiplicity of infection (MOI) of 2. ***p ≤ 0.001, Two-Way Anova with multiple comparisons. Values are expressed as mean ± SEM. Graphs show combined values from three independent experiments.

**At the exception of SYTOX kinetics A and B (beyond of the current Scopus), all data have been included in the revised manuscript.**

7. Figure 3A – are these capase-1 single KO or Casp1/11 DKO? If they are caspase-1/11 DKO neutrophils, NLRC4 will serve as a better control, since Casp11 is known to trigger NETosis.

The data are from single Caspase-1 KO neutrophils. The results of extracellular DNA release (NETs) in WT, Casp1KO and GSDMDKO neutrophils are very similar: There is no release of extracellular DNA when neutrophils are infected with *P. aeruginosa* pyroptotic strains PAO1^ΔExoS^ and PP34^ExoUS142A^. Regarding PP34 (NETotic strain) we observe almost a 100% extracellular DNA^+^ cells, independently of GSDMD or Caspase-1 expression. Regarding PAO1, we observe a mix phenotype. 20% of cells release DNA in Casp1/GSDMD-independent manner, whereas there is 20-25% cells that undergo pyroptosis (no extracellular DNA release, but Casp1 and GSDMD-dependent cell lysis **figures 1 and 2 in the manuscript**). Given that Caspase-11 also promotes GSDMD-dependent NETosis, we are confidents that the lack of involvement of GSDMD in P. aeruginosa-dependent NETosis is not driven by Caspase-11. Below are the quantifications of the experiments performed. Those quantifications have been included in the revised manuscript.

**Refer to Fig 3A in the manuscript.** Scanning electron microscopy (SEM) observations and quantifications of pyroptosis in WT, Casp1-/- and GsdmD-/- BMNs 3 hours after infection with P. aeruginosa strains PAO1, PAO1ΔExoS, PP34 and PP34ExoUS142A at an MOI of 10 (PAO1 strains) and 2 (PP34 strains). Images are representative of one experiment performed 3 times. Scale bars are directly indicated in the Figure. For quantifications, the percentage of cells exhibiting extracellular DNA was determined by quantifying the ratios of cells positives for extracellular DNA over the total of cells. 6-10 fields from n=3 independent experiments were analyzed. Values are expressed as mean ± SEM. ***p ≤ 0.001, Two-Way Anova with multiple comparisons.

**Those data have been included in the revised manuscript (Figure 3A).**

8. I am very confused by Figure 3. Line 284 ‘murine BMN with PA01, PA01ExoS-, PP34ExoU or PP34EoxU- strains and measured for the presence of NETs using SEM (Fig. 3A)’. However, I can’t seem to find the condition ‘PP34ExoU-‘ in Figure 3A. Did the authors mean PP34ExoUS142A instead? What is PP34ExoU? Does the strain normally express ExoU or is this introduced into the strain? If they naturally express ExoU, labelling it PP34 will suffice. Since the start of Figure 3 is confusing and poorly labelled, it is challenging to provide any useful comments for the rest of Figure 3.

We apologize for this. We tried to rename all our strains used and to combine our figure with more specific details to help the understanding as well as to be more rigorous about our writing of the results and referencing to the figures.

**Refer to Fig 3A in the manuscript.** Scanning electron microscopy (SEM) observations and quantifications of pyroptosis in WT, Casp1-/- and GsdmD-/- BMNs 3 hours after infection with P. aeruginosa strains PAO1, PAO1ΔExoS, PP34 and PP34ExoUS142A at an MOI of 10 (PAO1 strains) and 2 (PP34 strains). Images are representative of one experiment performed 3 times. Scale bars are directly indicated in the Figure. For quantifications, the percentage of cells exhibiting extracellular DNA was determined by quantifying the ratios of cells positives for extracellular DNA over the total of cells. 6-10 fields from n=3 independent experiments were analyzed. Values are expressed as mean ± SEM. ***p ≤ 0.001, Two-Way Anova with multiple comparisons.

9. The authors are proposing that neutrophil caspase-1 function is detrimental for host defence when infected with pyroptosis-inducing Pseudomonas, but offers no mechanistic insights into this observation.

Indeed, at this step we do not know if pyroptosis and/or IL1 cytokines from neutrophils contribute to the detrimental function we observe upon P. aeruginosa. We have included some putative leads of explanation in the discussion by referring to the articles from Faure et al, and from the Prince lab about the role of inflammasome-derived IL-1cytokines on the susceptibility of animals to P. aeruginosa. In addition, we also discuss the possibility that pyroptosis of neutrophils might also be a sterile and inefficient process at trapping P. aeruginosa as this particular bacterium has previously been shown to be able to direct inject Flagellin through the plasma membrane of cells, in absence of phagocytosis.

Hence, we discussed this in the discussion part as follows:

“Our study initially aimed at determining if neutrophil resistance to undergo Caspase-1-dependent pyroptosis can be overcome upon bacterial infection. Screening various inflammasome-activating bacteria [5,28,29], we found that P. aeruginosa successfully trigger murine and human neutrophil pyroptosis through the engagement of the fully competent canonical NLRC4 inflammasome, a process that requires T3SS-dependent injection of bacterial Flagellin. Although those results show a clear ability of neutrophils to undergo Caspase-1-dependent pyroptosis upon P. aeruginosa infection, our investigations performed with mice specifically lacking Caspase-1 in the granulocyte compartment do suggest a minor contribution of Caspase-1 upon P. aeruginosa infection. Related to this, previous studies showed a robust contribution of NLRC4 at promoting P. aeruginosa spread in various organs, which suggests that macrophages or other NLRC4-expressing cells are stronger contributors of mouse susceptibility to P. aeruginosa [50,51]. Related to this, NLRC4-driven both IL-1β and IL-18 release upon P. aeruginosa infection has been linked to an altered IL-17 production and a defect of mice to efficiently control P. aeruginosa [50,51]. Here, we speculate that the contribution of neutrophils at releasing IL-1β might also help the deviated immune response of the host to P. aeruginosa, hence leading to a better survival of this bacterium in host tissues. Another guess relies on the preferred extracellular tropism of P. aeruginosa. Hence, one could speculate that T3SS-injected Flagellin directly through the neutrophil plasma membrane might trigger a sterile pyroptosis and favors P. aeruginosa escape from microbial trapping and/or killing [52].”

10. I recommend to pool data from different experiments to increase the robustness of the study

Accordingly, we pooled most of the experiments regarding Sytox incorporation, LDH and IL-1beta release from n=3 independent experiments, each time performed in triplicate.

For human, we present results from n=3 individual patients (three wells/patients/condition) with specific symbol for each patient.

Minor points

1. Neutrophils are only resistant to caspase-1 but not caspase-11-driven pyroptosis. For clarity and accuracy, this should be changed in the abstract, summary and introduction.

Accordingly, this has been modified directly in the abstract.

2. I am not sure if the requirement for GSDMD in ROS-driven NETosis still stands, given that a recent article from Dixit’s lab showed that LDC7559 inhibits ROS instead of GSDMD (PMID: 34320407). This should be clarified in the introduction.

We fully agree with our reviewer comment. We will include in the introduction the still ongoing and open question regarding the role of Gasdmerin-D on NETosis upon PMA exposure.

**Introduction of the manuscript.** “Depending on the initial trigger, various signaling pathways such as calcium fluxes [17,18], necroptosis-associated MLKL phosphorylation [21], ROS-induced Neutrophil protease release [15] or endotoxin-activated Caspase-11 [3,5,22] have been shown to induce NETosis. ROS- and Caspase-11-dependent NETosis have been shown to share the requirement for cleavage of the pyroptosis executioner Gasdermin D (GSDMD) by neutrophil serine proteases and Caspase-11, respectively [3,16]. Active GSDMD forms pores on PIP2-enriched domains of the plasma and nuclear membranes of neutrophils, which ensures both IL-1ß secretion [23–26] and osmotic imbalance-induced DNA decondensation and expulsion [3,16]. However, the link between ROS and Gasdermin-D-dependent NETosis requires more investigations as a recent study could show that a described GSDMD inhibitor, the LDC559, is actually a ROS inhibitor, but not a GSDMD inhibitor [27].”

3. Reference 30 suggest neutrophil undergo NLRP3 activation and NETosis during SARS-CoV-2 infection, not sterile inflammation.

We agree, we also have included SARS-CoV2 in this part.

4. Line 265 typo – Figure 2E not 3E

5. Line 272 and 276 typo – Fig 2F not 2D

6. There are a lot more typo mistakes in the manuscript, please fix them

We have fully checked, modified and proof-read the manuscript for all the typos issues. We hope, we could solve the majority of them in the modified version of the manuscript. We apologize in advance if some are still present despite multiple checks.

7. If PP34ExoU is used for most parts of the in vivo characterisation, the ability for them to trigger pyroptosis should be moved to the main figure, not the supplementary.

Accordingly, we also moved into the main panels the most relevant experiment performed with the pyroptotic strains PP34ExoUS142A and/or PP34deltaExoU.

Part III – Minor Issues: Editorial and Data Presentation Modifications

Please use this section for editorial suggestions as well as relatively minor modifications of existing data that would enhance clarity.

**Reviewer #1:**

Abstract: The authors mention arms race in the abstract but never again- please remove as there is no data supporting a role for this phenomenon in the host microbe arms race.

We have removed this sentence in the abstract from the revised manuscript.

The data presented do not show that Pad4 gets activated by GSDMD as stated in the abstract, please rephrase

We have rephrased it by saying that GSDMD is required for PAD4-driven Histone citrullination. In addition, we now included two novel experimental sets that aim at addressing the contribution of activated GSDMD vs the contribution of GSDMD-induced neutrophil lysis on PAD4-driven Histone citrullination and subsequent decondensation. Here, using Glycine as an osmoprotectant (avoid complete cell lysis, but still allows efficient inflammasome signaling with IL-1beta release) we could determine that Histone Citrullination occurs in a neutrophil lysis independent manner.

In addition, we could also show that chelating extracellular Calcium with EGTA completely abrogate GSDMD-driven Histone Citrullination and DNA decondensation, but not inflammasome activation and cell lysis.

Hence, we are confidents that GSDMD supports extracellular Calcium- and PAD4-induced Histone Citrullination and DNA decondensation, in a neutrophil lysis-independent manner.

**Those results have been included in the Supplemental 4 Figure of the manuscript.**

**From Supplemental Figure 4. Inflammasome signalling induce neutrophil DNA decondensation through Calcium-dependent, but cell lysis-independent, manner.**

A. Confocal microscopy observations and quantifications of human blood neutrophils infected for 3 hours with PAO1ΔExoS (MOI 10) or PP34ExoUS142A (MOI 2) in presence/absence of Caspase-1 inhibitor Z-YVAD (20µM) and harboring Citrullinated Histone 3 (H3-Cit), decondensed DNA and nuclear membrane (LaminB1). Nucleus (blue) was stained with Hoechst; Citrullinated Histone-3 is in red (anti H3-Cit); plasma membrane is in grey (WGA staining). Scale bar 10µm. Images are representative of one experiment performed three times with at least 150 neutrophils observed/ experiment.

B. Immunoblotting of Citrullinated Histone 3 (H3Cit), total Histone 3 and preformed and cleaved Gasdermin-D (p55/p30) in WT, Nlrc4-/- and GsdmD-/- BMNs treated with Ionomycin (10µM, 3 hours) or infected for 3 hours with PAO1, PAO1ΔExoS, PAO1ExsA- (MOI 10) or with PP34 , PP34ΔExoU (MOI 2). Immunoblots show combined lysates and supernatants from one experiment performed three times.

C. Confocal microscopy observations and quantifications of WT and GsdmD-/- BMNs infected for 3 hours with P. aeruginosa pyroptotic strain PAO1ExoUS142A (MOI 2) in presence/absence of EGTA (10mM) and harboring Citrullinated Histone 3 (H3-Cit) and decondensed DNA. Nucleus (blue) was stained with Hoechst; Histone-3 Citrullination is in red (Anti-H3Cit staining). For quantifications, the percentage of cells positives for H3Cit (H3-Cit+) and decondensed DNA was determined by quantifying the ratios of cells positives for H3Cit and decondensed DNA over the total amount of cells. At least 6 fields from n=3 independent experiments were analyzed. Values are expressed as mean ± SEM. ***p ≤ 0.001, Two-Way Anova with multiple comparisons.

D. Measure of cell lysis (release of LDH) and IL-1β release in WT or GsdmD-/- BMNs infected for 3 hours with PP34ExoUS142A (MOI2) or PAO1ΔExoS (MOI 10) in presence/absence of EGTA (10mM). ***p ≤ 0.001, Two-Way Anova with multiple comparisons.

Values are expressed as mean ± SEM. Graphs show combined values from three independent experiments.

E. Confocal microscopy observations and quantifications of WT and GsdmD-/- BMNs infected for 3 hours with P. aeruginosa pyroptotic strain PAO1ExoUS142A (MOI 2) in presence/absence of Glycine (5mM) and harboring Citrullinated Histone 3 (H3-Cit) and decondensed DNA. Nucleus (blue) was stained with Hoechst; Histone-3 Citrullination is in red (Anti-H3Cit staining). For quantifications, the percentage of cells positives for H3Cit (H3-Cit+) and decondensed DNA was determined by quantifying the ratios of cells positives for H3Cit and decondensed DNA over the total amount of cells. At least 6 fields from n=3 independent experiments were analyzed. Values are expressed as mean ± SEM. ***p ≤ 0.001, Two-Way Anova with multiple comparisons.

F. Measure of cell lysis (release of LDH) and IL-1β release in WT or GsdmD-/- BMNs infected for 3 hours with PP34ExoUS142A (MOI2) or PAO1ΔExoS (MOI 10) in presence/absence of Glycine (5mM). ***p ≤ 0.001, Two-Way Anova with multiple comparisons.

Values are expressed as mean ± SEM. Graphs show combined values from three independent experiments.

Introduction: Line 149: Data by Rauch et al 2016 suggest Pseudomonas also activates the Naip6-Nlrc4 inflammasome

Thank you pointing this to us. We have modified the text accordingly.

Statistics: T- test does not seem the appropriate test for several of these experiments, where multiple groups are being compared.

When required (multiple groups), we used a 2-way anova test with multiple comparisons.

Figure legends: sometimes the authors state in the figure legends: one experiment performed three times. I assume they mean “one experiment representative of three independent experiments” please clarify.

Indeed, it is one experiment representative of 3 independent experiments. However, according to reviewer suggestions, we now show pooled experiments from 3 independent experiments, each performed in triplicate. This has been specified in each figure legend.

**Editorial:**

Multiple places in manuscript: It is spelled Hoechst dye.

This has been corrected in the revised manuscript.

Line 165: I don’t believe WT humans is an appropriate term

This has been modified accordingly: we now use the term “healthy patients” or “healthy donors”.

205-209: Salmonella infection is referenced in 1B but not shown? Please show the data or remove in the text

This has been removed as it was a remnant from a previous draft of the manuscript.

214-215 Flagellin is not a toxin please rephrase

This has been rephrased accordingly in the text

220-223: The last panel in 1C suggests, other than discussed in the text, that Flagellin is the sole inflammasome activator as the difference between WT and Casp1 is lost in the FliC- strain. A phenotype in the T3SS system deficient strain can be explained that without the secretion system, no flagellin will leak into the cytosol.

We fully agree and now included description of this in the result part. The ExsA mutant was used as a control of the lack of NLRC4 response to P. aeruginosa.

“P. aeruginosa T3SS-mediated injection of Flagellin and toxins (ExoS, U, T, Y) play a major role in its virulence. To determine the role of these virulence factors in P. aeruginosa-induced neutrophil pyroptosis, we infected murine BMNs with WT P. aeruginosa PAO1 or mutant strains that are deficient in expression of individual toxins (PAO1ΔExoS, PAO1ΔExoT, PAO1ΔExoY); deficient for the expression of Flagellin (PAO1ΔFliC); or for the T3SS (PAO1ΔExsA) (Fig. 1C, S1B Fig). We parallelly measured the ability of WT and Casp1-/- neutrophils infected with these strains to undergo cell lysis (LDH release), to promote IL1β release, and to exhibit plasma membrane permeabilization (SYTOX Green incorporation) (Fig. 1C, S1B Fig). Our results show that P. aeruginosa strains lacking expression of T3SS or Flagellin were unable to promote robust Caspase-1-dependent neutrophil lysis, IL-1β release and plasma membrane permeabilization, suggesting that T3SS and Flagellin are major effectors of Caspase-1-driven neutrophil death (S1B Fig, Fig. 1C). Flagellin deficiency modifies the physical recognition and phagocytosis of P. aeruginosa by macrophages and neutrophils [45,46] (S1C Fig.), which could indirectly alter the amount of Flagellin injected into host target cells. To ensure similar phagocytosis/recognition of Pseudomonas aeruginosa strains by neutrophils, we genetically invalidated the expression of Flagellin motors (namely MotABCD) in a WT PAO1 and Flagellin-deficient (PAO1ΔFliC) genetic background. Both PAO1ΔMotABCD and PAO1ΔMotABCD/ΔFliC showed similar neutrophil uptake after 45 minutes of infection (S1C Fig.), which allowed evaluating the direct importance of Flagellin in P. aeruginosa-driven Caspase-1-dependent neutrophil pyroptosis. Kinetic analysis of neutrophil lysis (LDH release) and IL-1β production in WT and Casp1-/- neutrophils showed that PAO1ΔMotABCD, but not PAO1ΔMotABCD/ΔFliC, induced Caspase-1-dependent neutrophil lysis and IL-1β release over time (S1D Fig.), suggesting that Flagellin plays a major role in P. aeruginosa-induced neutrophil pyroptosis.”

Regarding the very low role of T3SS components from P. aeruginosa (e.g. needle) at triggering NLRC4-depndent neutrophil pyroptosis, we think that we are not sensitive enough to measure the impact of the T3SS needle of *P. aeruginosa* on neutrophil pyroptosis (LDH release). We now discuss this more in depth in the result section. We also have included new data that support the prominent role of Flagellin at activating the neutrophil NLRC4 inflammasome. However, our results do not state for a lack of importance or effect of the T3SS components on the NLRC4 inflammasome in neutrophils. We think that, using LDH assays, we are not sensitive enough to measure their respective importance in neutrophils.

246: there is still a significant difference between infected and uninfected Casp1-/- at later timepoints in S1D:, so please rephrase line “in a fully Caspase-1 and GSDMD-dependent manner.” to a more appropriate statement

This has been modified by stating that Caspase-1 and GSDMD plays a major role at promoting neutrophil lysis.

Line 265: reference to fig3E I believe this reference S2C?

Indeed, we realized that we mislabeled many of the figures, this has been checked carefully and now all figures are rightly referenced.

Line 272: refence to 2F instead of 2D

Indeed, we realized that we mislabeled many of the figures, this has been checked carefully and now all figures are rightly referenced.

Line 304: “neither PAO1ExoS- nor PP34ExoU- induced Histone release” As far as I can tell there is no ExoU- data shown in these figures, do the authors mean ExoUS142A?

Indeed, we meant ExoUS142A. This has been corrected. We now provide also various supports (schemes, references in the manuscript) about the Pseudomonas strains that trigger pyroptosis or not in neutrophils.

Line 319 “Histone3-Citrullination in a NLRC4-, GSDMD- and CASP-1-dependent manner “- there is still H3-Cit bands visible in S3E in Gsdmd and Nlrc4-knockouts, just weaker, and no Casp1 data is shown, please rephrase ethe statement to reflect that

Accordingly, we have re-written this part in the manuscript:

“In order to further validate these observations, we performed immunoblotting against citrullinated histones in WT, Nlrc4-/- and GsdmD-/- neutrophils infected with the pyroptotic P. aeruginosa strains PAO1, PAO1ΔExoS, PP34ΔExoU or with the NETotic strain PP34. As control, we used Ionomycin, a known inducer of histone citrullination-driven NETosis. We observed that Ionomycin triggered NLRC4-independent histone citrullination in neutrophils (S4B Fig.). To the contrary, pyroptotic strains of P. aeruginosa PAO1ΔExoS, PP34ΔExoU and to a lower extend PAO1 all triggered NLRC4- and GSDMD-dependent histone citrullination (S4B Fig.). We noticed that in Nlrc4-/- and GsdmD-/- neutrophils, citrullination was not fully abrogated, suggesting that minor alternative pathways might also promote histone citrullination upon infection with P. aeruginosa pyroptotic strains (S4B Fig.).”

320: Human blood neutrophils not in S3C but D

This has been modified and corrected in the manuscript

370-73: Figures 4D+G: IL-1 levels in the lungs of these mice could just be decreased because of lower bacterial levels, please discuss that in the description of the data.

This has been directly included in the description of the data part in the revised version of the manuscript:

“To the contrary, MRP8^Cre+^*Casp1^flox^* mice infected with PAO1, showed a slight but significant improved bacterial elimination in Bronchoalveolar Fluids (BALF) and lungs, a phenotype that was further amplified upon infection with PAO1^ΔExoS^ and PP34^ExoUS142A^ pyroptotic strains (**Fig. 5 B-G**). Furthermore, IL-1β levels in BALFs were decreased in MRP8^Cre+^*Casp1^flox^* mice infected with PAO1, PAO1^ΔExoS^ and PP34^ExoUS142A^. Although we cannot exclude that lower bacterial loads in MRP8^Cre+^*Casp1^flox^* mice could influence the levels of IL-1β measured in BALFs, those results suggest that neutrophil Caspase-1 is also a contributor of IL-1β production upon PAO1, PAO1^ΔExoS^ and PP34^ExoUS142A^ infections (**Fig. 5 D, G**). Altogether, our results highlight that neutrophil Caspase-1 contributes to both IL-1β release and mouse susceptibility to several *P. aeruginosa* strains (**Graphical Abstract**).”

374-75: The claim about spread not valid based on the data shown as the authors only examine lung tissue but not spread to other organs, please remove.

We fully agree, and removed this term.

Figure 1C: please label the X-axis (I assume its minutes)

Axis have all been labeled accordingly

Figure 3: PAD4 has been shown to be required for NETosis in papers the author cite- so the figure title "Caspase-1-induced neutrophil pyroptosis promotes PAD4-dependent incomplete NETosis" is misleading as it suggests that pAD4 is causing incomplete NETosis, which is not what the authors show

We have replaced this sentence but also the terms NETosis and/or incomplete NETosis in the manuscript for more accuracy and clarity.

We now refers to “Caspase-1-induced pyroptosis” or “pyroptosis” to avoid further confusion.

Discussion: Please discuss why PAD4 in this system would lead to only H3 citrunilation but not NETosis as observed by others.

We discuss this in the discussion part as follow:

“Although NLRC4 activation leads to Caspase-1 dependent neutrophil pyroptosis, in a pathway similar to what was previously reported in macrophage [39,48], neutrophil pyroptosis exhibits a unique feature characterized by nuclear membrane rupture, DNA decondensation and expansion within cell cytosol. One key enzyme responsible for this morphological characteristic of neutrophil pyroptosis is the Protein arginine deiminase 4 (PAD4), activated by Calcium fluxes. Indeed, similar to the process induced by various NETosis inducers, Caspase-1 also promoted Calcium- and PAD4-dependent Histone Citrullination, which stimulated DNA relaxation and release from the nucleus but, surprisingly, not its extracellular expulsion. Why upon Caspase-11 [3], MLKL [21], NADPH [17] or NE/CatG/Pr3 [15] stimulation but not upon Caspase-1 activation neutrophils generate two different types of DNA structures remains yet to be investigated. Recently, Thiam et al., [18] observed that pharmacological stabilization of F-actin allowed the development of this “incomplete NETosis” upon Ionomycin-exposure. Interestingly, neutrophil elastase has also been shown to degrade actin [58], hence ensuring complete NETosis process. This, suggests that efficient actin degradation and/or depolymerization may be an essential player of extracellular DNA release, which would imply that the final step of NETosis might actually be a cell-regulated process involving various controllers [14,20]. Interestingly, Chen and colleagues recently found that upon infection with Yersinia, murine neutrophils induce a pyroptotic program that involves virulence-inhibited innate immune sensing, hence promoting RIPK1-induced Caspase 3-dependent Gasdermin E cleavage and activation and pyroptosis [59], a process that does not trigger NETosis. This further suggests that multiple Gasdermins can trigger neutrophil pyroptosis through multiple molecular pathways and promote different morphological outcomes of neutrophils.”

Please discuss the absence of a Caspase-11 phenotype with this bacterium

We now discuss this aspect in the discussion part as follow:

“Related to this, studies showing that activation of the neutrophil NLRC4 inflammasome by S. Typhirmurium or the opportunistic pathogen B. thaïlandensis did not promote canonical pyroptosis. Instead those bacteria triggered Caspase-11-dependent pyroptosis. This suggests that, in absence of efficient canonical inflammasome-driven pyroptosis, Caspase-11 might compensate at triggering neutrophil pyroptosis upon Gram-negative bacterial infections. Here, the lack of Caspase-11-dependent pyroptosis in neutrophils upon P. aeruginosa infection suggests that NLRC4 inflammasome-induced neutrophil pyroptosis upon P. aeruginosa infection is well effective and sufficient in this context.”

**Reviewer #2: (No Response)**

Reviewer 3

****Part I - Summary

Please use this section to discuss strengths/weaknesses of study, novelty/significance, general execution and scholarship.

A number of studies have previously shown that neutrophils do not undergo pyroptosis in response to a range of canonical inflammasome stimuli (e.g. PMID: 25043180; PMID: 30089254; PMID: 25609842; PMID: 26877061) whereas non-canonical inflammasome agonists (i.e. cytosolic Gram-negative bacteria/LPS) induce caspase-11/GSDMD-dependent NETosis (PMID: 30143554). In this study, the authors set out to explore pyroptosis resistance in neutrophils in further detail, specifically in the context of canonical inflammasome-activating bacteria.

The main message of the paper is that Pseudomonas aeruginosa drives NLRC4-caspase-1 dependent pyroptosis in neutrophils. This is perhaps misleading given that the WT Pseudomonas aeruginosa strain used in the study is actually a stronger inducer of NETosis, which has been published previously (e.g. PMID: 30930127), whereas strains lacking ExoS or ExoU drive caspase-1-dependent pyroptosis and ‘incomplete NETosis’. Thus, the novelty of this finding is not completely convincing, nor is the physiological relevance given that physiological Pseudomonas aeruginosa strains express either ExoU or ExoS. Nonetheless, these experiments and findings are interesting but perhaps the overall message of the study could be modified.

Overall, the authors’ work relating to the pyroptotic cell death and IL-1 production is interesting, particularly since neutrophils tend to die in response to most stimuli (microbes, crystals, mitogens), rather than being “resistant” to pyroptosis. However, the NET-related angle needs to be refined as much existing literature is ignored. Branzk et al. (PMID: 25217981) have previously shown that small microbes do not induce chromatin decondensation due to sequestration of NE to the phagosome. Citrullination still occurs in the presence of NE inhibitors. Bacteria clusters however were good inducers of NETosis. The mechanism predicted that chromatin decondesation would occur in response to biofilms which was subsequently demonstrated in a paper from Paul Kubes on P. aeruginosa (PMID: 30930127). Hence, if the authors wish to study the role of Nod-like receptors and caspases in NETosis then they should use P. aeruginosa biofilms. Otherwise, what they are studying is the same cell death programme induced during NETosis but accompanied by failure of potent chromatin chromatin decondensation with chromatin hat does not exceed the PM boundaries which can be seen in response to many bacteria and yeasts. I guess failed NETosis might be a better word, but it is clear that NETosis is pyroptosis+chromatin deondensation, whereas what the authors study is pyroptosis+minimal chromatin decondesation. Nevertheless, the failure in NETosis may be either due to NE translocation which has been linked to GSDMD or failure to efficiently lyse the PM. These concepts could be clarified here and help focus the manuscript on novel mechanistic aspects of these responses.

We thank our reviewer for his/her very insightful suggestions and advices on the study. We have tried answering them in our revised version of the manuscript. We now present results as well as interpretations through the pyroptotic angle and did not link it to NETosis or incomplete NETosis to avoid overstatements and further confusion/misleading interpretations.

Part II – Major Issues: Key Experiments Required for Acceptance

Please use this section to detail the key new experiments or modifications of existing experiments that should be absolutely required to validate study conclusions

1) Neutrophils have been shown to be resistant to cell lysis (no LDH release) following NLRC4 inflammasome activation in response to S. typhurium or B. thailandensis (PMID: 25043180; PMID: 32726630) and indeed this is mentioned in the introduction. However, S. typhurium or B. thailandensis appear to induce LDH release (despite not being caspase-1 dependent) in neutrophils in Fig 1A. Please could the authors provide potential explanation(s) for the disparities seen between the data in these in vitro experiments and exisiting literature. Specifically, experiments could be done to delineate specifically what form of cell death these stimuli induce, e.g. looking at markers of necroptosis, necrosis, or by using different caspase inhibitors.

We fully agree with our reviewer interrogation. This is indeed a critical question we have also tried answering, unfortunately without full success yet. At this step, we cannot provide a fully satisfactory and experimentally relevant answer as we are still trying to understand why in our experimental conditions (and others), S. typhimurium and B. thailandensis induce neutrophil lysis, yet in a Caspase-1-independent manner in murine neutrophils.

Importantly, different people of the group (R. Planes, K. Santoni, M. Pinilla, L. Gorse, E. Meunier) have performed those experiments, which suggests that this is not experimenter-driven observation. In addition, the group of Mohamed Lamkanfi (University of Ghent, Belgium) has also done similar observations in a complementary study performed in collaboration with our group (currently in revisions in another journal), which also supports that those observations are not geographically-driven.

We speculate that the MOI, the way bacterial subcultures were done (no more than OD 600 0.8 in order to avoid decreased expression of the T3SS SPI-1 from Salmonella), neutrophil priming or not (in both studies cited by our reviewer neutrophils were primed either with LPS or LPS+IFNgamma; our study does not use primed neutrophils) might all influence the neutrophil ability to 1/ efficiently kill bacteria and 2/ lyse or not.

In order to determine what were the general pathways important for neutrophil lysis induced by S. Typhimurium and B. thailandensis, we used human blood neutrophils (unprimed) treated with a panel of known and characterized inhibitors of various cell death processes, including Apoptosis/Lytic Apoptosis (Z-DEVD:Casp3/7 inhibitor, ZIETD:Casp8 inhibitor), Caspase-1-driven pyroptosis (YVAD), Caspase-4/5-driven pyroptosis (Z-LEVD), Necroptosis (Nec1s inhibitor) or ferroptosis (Ferrostatin-1 inhibitor).

An extremely disturbing observation we did is that, contrary to murine neutrophils that hardly lyse through Caspase-1 upon *S*. Typhimurium or *B. thailandensis* infection, **human blood neutrophils undergo efficient Caspase-1-driven pyroptosis in response to those bacteria (figure below)**.

This surprising observation is currently under investigation in the group in order to determine the major differences between murine and human neutrophils that control pyroptosis induction or not for the same microbial pathogen.

Here, we ask our reviewer and editor allowing us to not include those results in the revised manuscript as 1/ this is currently under investigations and requires further experimental validations.

**Figure**. Measure of cell lysis (release of LDH) in human blood neutrophils infected for 3 hours with *S.* Typhimurium (strain SL1344, MOI 10) or *B. thailandensis* (strain E264, MOI 30) in presence/absence of various Caspase inhibitors, Z-VAD (pan Caspase, 20µM), Z-YVAD (Casp1 inhibitor, 40µM), Z-DEVD (Casp3 inhibitor, 40µM), Z-IETD (Casp8 inhibitor, 40µM), Z-LEVD (Casp4/5 inhibitor, 40µM), Necroptosis inhibitor Necrostatin1s (Nec1s, 40µM) or Ferroptosis inhibitor Ferrostain-1 (Fe1, 20µM). ***p ≤ 0.001, Two-Way Anova with multiple comparisons. Values are expressed as mean ± SEM. Graphs show values from three independent experiments with one different donor each time.

2) Fig. 1D. A stain for the total amount of cells should shown as well. (Hoechst, DAPI etc).

We now provide a stain for the total amount of cells.

**Refers to Fig1D of the revised manuscript.** Fluorescent microscopy images using SYTOX Green incorporation in WT or Casp1-/- BMNs infected with Pseudomonas aeruginosa PAO1 or its isogenic mutant lacking T3SS-derived toxin ExoS (PAO1ΔExoS). Hoechst (Blue, DNA), Sytox Green (DNA from neutrophils with permeabilized membranes, Green). Scale Bar: 50µm

3) Whilst DNA decondensation is assessed by IF with DAPI in Fig 3B and Supp Fig 3B+C, it could benefit from some quantification of DNA area to measure differences between the conditions.

We have performed quantifications of DNA area ratios (Fluorescence experiments) or ratio of cells with extracellular DNA over total amount of cells (SEM experiments) as described below (Software used ImageJ).

A. Scanning electron microscopy (SEM) observations and quantifications of pyroptosis in WT, Casp1-/- and GsdmD-/- BMNs 3 hours after infection with P. aeruginosa strains PAO1, PAO1ΔExoS, PP34 and PP34ExoUS142A at an MOI of 10 (PAO1 strains) and 2 (PP34 strains). Images are representative of one experiment performed 3 times. Scale bars are directly indicated in the Figure. For quantifications, the percentage of cells exhibiting extracellular DNA was determined by quantifying the ratios of cells positives for extracellular DNA over the total of cells. 6-10 fields from n=3 independent experiments were analyzed. Values are expressed as mean ± SEM. ***p ≤ 0.001, Two-Way Anova with multiple comparisons.

**Refers to Fig 3C of the manuscript.** Representative time lapse fluorescence microscopy images and quantifications of ASC-Citrine murine BMNs infected with the NETotic strain PP34 or the pyroptotic strain PP34ExoUS142A (MOI 2) for 9 hours (540 minutes). Nucleus (blue) was stained with Hoechst; ASC is in yellow (ASC-Citrine); plasma membrane is in green (WGA staining); plasma membrane permeabilization is stained in red (cell impermanent DNA dye Propidium Iodide, PI). Images are representative of one movie out of three independent movies. For quantifications, the percentage of cells harboring decondensed DNA and/or extracellular decondensed DNA was determined by quantifying the ratios of cells with decondensed DNA (area surface) or cells with decondensed DNA crossing WGA staining (DNA area surface outside from plasma membrane) over the total amount of cells. At least 6 fields containing each 20-30 cells were quantified. Scale bar 10µm. ***p ≤ 0.001, T-test with Bonferroni correction.

4) The images in Fig 3D are slightly confusing. An alternative experiment to show that NET DNA is not being released in response to the pyroptotic mutants the authors could measure DNA area (SYTOX signal) beyond the membrane (WGA), as similar experiments have been conducted in Fig 4A.

Accordingly, we have performed quantifications of DNA area ratios as described below (Software used ImageJ).

**Refers to Fig 3C of the manuscript.** Representative time lapse fluorescence microscopy images and quantifications of ASC-Citrine murine BMNs infected with the NETotic strain PP34 or the pyroptotic strain PP34ExoUS142A (MOI 2) for 9 hours (540 minutes). Nucleus (blue) was stained with Hoechst; ASC is in yellow (ASC-Citrine); plasma membrane is in green (WGA staining); plasma membrane permeabilization is stained in red (cell impermanent DNA dye Propidium Iodide, PI). Images are representative of one movie out of three independent movies. For quantifications, the percentage of cells harboring decondensed DNA and/or extracellular decondensed DNA was determined by quantifying the ratios of cells with decondensed DNA (area surface) or cells with decondensed DNA crossing WGA staining (DNA area surface outside from plasma membrane) over the total amount of cells. At least 6 fields containing each 20-30 cells were quantified. Scale bar 10µm. ***p ≤ 0.001, T-test with Bonferroni correction.

5) In Fig 3D, there is an additional GSDMD cleavage product (below the p30 cleavage band) that is only present in response to the PP34 ExoU+ (NETotic) strain. Could this fragment be responsible for driving NETosis in response to the PP34 ExoU+ strain, given that it does not appear in response to the ExoU pyroptotic mutant?

6) The combination of pores formed by these fragments may lead to further membrane instability leading to total cell lysis and thus NET DNA release, whereas in response to the pyroptotic mutant the level of GSDMD pore formation may not be sufficient to permit NET DNA release. The generation of this fragment also appears to be NLRC4-independent as it is still produced by NLRC4-/- neutrophils in response to ExoU pyroptotic mutant. Alternatively, differences may lie with NE translocation since GSDMD has been implicated in NE translocation to the nucleus. IF staining for CitH3/MPO/NE/DAPI and WB for histones and citrullinated H3 will be useful to distinguish between these possibilities and help understand the state of these cells. P. aureus biofilm induced neutrophils could also be used for comparison here and potentially in IL-1b and LDH release experiments, depending on where the authors wihs to go with the study.

**Questions 5 and 6:**

Indeed, we also wondered about the putative function of this intermediate fragment of GSDMD upon PP34 infection. However, infection of GsdmD-/-, NE-/-CatG-/-Pr3-/- or Gp91phox-/- neutrophils with PP34 (NETotic strain) did not show any defect in cell lysis or DNA decondensation or extracellular DNA production (**cf Figure below A, B, E, F**). What we observed is that PP34 infection leads to PAD4-dependent Histone Citrullination and subsequent DNA decondensation and expulsion from the cell (NETs) (**cf figure below** **C, D**). Finally, the pyroptotic P. aeruginosa strain PP34ExoUS142A triggers cell lysis, IL1beta release and intracellular DNA decondensation in a GsdmD-dependent manner but NE/CatG/Pr3-independent manner (**cf figure below G, H**)

Hence, here, we cannot say that this fragment of GSDMD is responsible or participate at driving NETosis in this specific context. A possibility to us is that upon NETosis induced by PP34, a significant amount of Serine Proteases such as NE, CatG and Pr3 are released to the host cell cytosol and can cleave at different sites GSDMD, hence generating those intermediate fragments. However, given that GSDMD deficiency in neutrophils does not influence any of the studied aspects of neutrophils lysis (LDH release, DNA decondensation, extracellular DNA access), we hypothesize that this might occur downstream of cell lysis induced by PP34 strain.

Regarding the suggested biofilm experiments, indeed, this has been nicely addressed by the Paul Kubes **lab** that could show a very important function of ExoS-expressing P. aeruginosa at promoting corneal neutrophil NETosis in mice models. This is a very important stone on which to build future studies on the role of biofilms on neutrophil lysis and NETosis. However, here, to avoid further confusion, we mostly aim at focusing on Caspase-1-driven pyroptosis in neutrophils as a proof of principle that even individual bacteria can promote Caspase-1-dependent neutrophil lysis (here referred to as pyroptosis).

**All results related to pyroptosis have been included in the revised version of the manuscript.**

**Regarding the data involving PP34 (NETotic strains), we wish to not include them in the current manuscript as 1/ they fall too far from the pyroptosis Scopus of this study and 2/ we are currently trying to determine the mechanisms by which ExoU induces such a potent NETosis.**

A. Measure of cell lysis (release of LDH) and IL-1β release in WT, Casp1-/-, Casp11-/-, Casp1-/-Casp11-/- and GsdmD-/- murine Bone Marrow Neutrophils (BMNs) infected for 3 hours with Pseudomonas aeruginosa PP34 strain or its isogenic mutant PP34ΔExoU at a multiplicity of infection (MOI) of 2. ***p ≤ 0.001, Two-Way Anova with multiple comparisons. Values are expressed as mean ± SEM. Graphs show combined values from three independent experiments.

B. Measure of cell lysis (release of LDH) in WT, Gp91phox-/-, GsdmD-/-, NE-/-CatG-/-Pr3-/- and Pad4-/- murine Bone Marrow Neutrophils (BMNs) infected for 2 hours with Pseudomonas aeruginosa PP34 strain at a multiplicity of infection (MOI) of 2. Values are expressed as mean ± SEM. Graph shows combined values from three independent experiments.

C. Immunoblotting observation of Histone 3, Histone 3-Cit, MPO in cellular extracts and in the supernatant from WT, Nlrc4-/- and NE-/-CatG-/-Pr3-/- murine BMNs infected with P. aeruginosa NETotic strain PP34 or pyroptotic strain PP34ExoUS142A (MOI 2) for 3 hours. Immunoblot shows one experiment performed two times.

D. Confocal microscopy observations and quantification of WT or Pad4-/- murine BMNs infected for 2 hours with P. aeruginosa PP34 (MOI 2) in presence/absence of the ExoU inhibitor MAFP (20µM) and harboring Histone3-Citrullination (H3-Cit), decondensed DNA (Hoechst) and plasma membrane (WGA). Nucleus (blue) was stained with Hoechst; H3-Cit is in Red; Plasma membrane is in grey (WGA). Scale bar 10µm. Images are representative of one experiment performed three times with at least 6-10 fields neutrophils observed/ quantified for H3-Citrullination and DNA decondensation. ***p ≤ 0.001, Two-Way Anova with multiple comparisons. Values are expressed as mean ± SEM.

E, F. Scanning electron microscopy (SEM) and confocal observations of NETosis in WT, Casp1-/-, GsdmD-/- and NE-/-CatG-/-Pr3-/- BMNs 3 hours after infection with P. aeruginosa PP34 at an MOI of 2. Images are representative of one experiment performed 3 times. Nucleus (blue) was stained with Hoechst; Plasma membrane is in grey (WGA).

G. Confocal microscopy observations and quantifications of WT and NE-/-CatG-/-Pr3-/- BMNs infected for 3 hours with P. aeruginosa pyroptotic strain PP34ExoUS142A (MOI 2) and harboring decondensed DNA and plasma membrane (WGA). Nucleus (blue) was stained with Hoechst; plasma membrane is in grey (WGA staining). For quantifications, the percentage of cells with nuclear DNA was determined by quantifying the ratios of cells positives for decondensed DNA on nuclear DNA. 6 fields from n=3 independent experiments were analyzed. Scale bar 30µm. ***p ≤ 0.001, T-test with Bonferroni correction.

H. Measure of cell lysis (release of LDH) and IL-1β release in WT, NE-/-CatG-/-Pr3-/- and GsdmD-/- murine Bone Marrow Neutrophils (BMNs) infected for 3 hours with Pseudomonas aeruginosa pyroptotic strain PP34ExoUS142A (MOI 2). ***p ≤ 0.001, Two-Way Anova with multiple comparisons. Values are expressed as mean ± SEM. Graphs show combined values from three independent experiments.

7) As a general comment, the writing of the manuscript needs major improvement. Moreover, several errors appear in the labelling of graphs/reference to figures in the text making it hard to properly understand the data.

We apologize for this and have now sent our manuscript for complete English proof-reading.

In addition, we also performed a complete check of figure and reference citation. We also provide some nomenclature for efficient reading and understanding of the different P. aeruginosa strains that promote pyroptosis and those that do not promote Caspase-1-depndent pyroptosis in neutrophils.

Part III – Minor Issues: Editorial and Data Presentation Modifications

Please use this section for editorial suggestions as well as relatively minor modifications of existing data that would enhance clarity.

•	Throughout the manuscript the authors use multiple t-tests with Bonferroni correction to compare more than two sets of data. Could the authors comment on why they have chosen this statistical test rather than ANOVA? Also, can the authors make it clear in the figure legend what groups are being compared. It would be good not only to see the statistical differences between groups, but also between treatments within the same group, for which a two-way ANOVA could be used.

Initially, we only compared treated WT vs treated KO. However, in order to make things more accurate, we now included a full analysis between different groups using a Two way anova test with multiple comparisons. We also directly labelled in figures what were the groups compared.

•	The strains used for the microbes in 1A are not stated in the figure legend nor the methods and should be included. For example, is the P. aeruginosa strain used in 1A the PAO1 strain used in the rest of the manuscript?

We apologize for the lack of clarity. The specific strains used have been precised and defined in all relevant parts of the manuscript.

•	In Fig 1A, there is a high level of IL-1b release in response to S. aureus in both WT and Casp-1-/- neutrophils. How do the authors anticipate that IL-1b is being cleaved into its mature form in the absence of caspase-1 here? Is this actually proIL-1b that has been released into the supernatant as a consequence of cell lysis?

Indeed, this is an intriguing point raised by our reviewer. At this step, we do not have yet tested if the released IL-1beta is in its active form or under the pro-form. Relying on the literature, we speculate that some S. aureus factors might directly be able to cleave pro-IL-1beta in its mature form upon neutrophil lysis.

This has been documented for protease secreted by S. epidermidis (<https://www.sciencedirect.com/science/article/abs/pii/S0022202X22003062>). Should S. aureus also performs such process is a possibility that we do not exclude, mostly because it expresses and releases a large panel of proteases such as Staphopains or the serine protease Spl (<https://www.ncbi.nlm.nih.gov/pmc/articles/PMC5061998/>).

In addition, such process of bacterial protease-driven IL-1beta cleavage and maturation has also be described for LasB serine protease released by P. aeruginosa (<https://www.ncbi.nlm.nih.gov/pmc/articles/PMC7511813/>) or for SpeB protease secreted by S. pyogenes (<https://www.ncbi.nlm.nih.gov/pmc/articles/PMC5358671/>).

Another explanation might also lie on a previous study that could show a Casp1-independent ability of Neutrophil to process IL-1beta in neutrophils infected with S. aureus (<https://www.ncbi.nlm.nih.gov/pmc/articles/PMC6394835/>). In this study the authors showed that Neutrophil Elastase could promote IL-1beta processing.

Finally, upon neutrophil lysis induced by S. aureus another possibility is that this lysis does not lead to IL-1beta processing but still promote pro-IL-1beta release.

We are sorry not being able answering experimentally this question for now. We are currently trying to determine the biological activity of S. aureus-induced Caspase-1-independent IL-1 release by immunoblotting and using HEK-IL-1R reporter cells lines.

•	In lines 205-209, the authors state that S. typhurium induces IL-1b release and cell lysis in human blood neutrophils. However this data cannot be seen in the figure and should be added.

Indeed, this was a remnant of a previous draft. We apologize for it.

As accidentally left in the draft of our previous manuscript, in human neutrophils Salmonella and B. thailandensis reproducibly trigger Caspase-1-dependent pyroptosis of neutrophils. This is in contrast to murine neutrophils where Caspase-1 is not a major player of neutrophil lysis upon both bacterial infections. This surprising observation is currently under investigation in the group and in collaboration with another lab in the frame of a complementary study that aims at determining the major differences between murine and human neutrophils that control pyroptosis induction or not for a given microbial pathogen.

Hence, here we ask our reviewer and editor allowing us to not include those results in the revised manuscript as we think that this is not the scope of the current study and would bring confusion at this step.

**Figure**. Measure of cell lysis (release of LDH) in human blood neutrophils infected for 3 hours with *S.* Typhimurium (strain SL1344, MOI 10) or *B. thailandensis* (strain E264, MOI 30) in presence/absence of various Caspase inhibitors, Z-VAD (pan Caspase, 20µM), Z-YVAD (Casp1 inhibitor, 40µM), Z-DEVD (Casp3 inhibitor, 40µM), Z-IETD (Casp8 inhibitor, 40µM), Z-LEVD (Casp4/5 inhibitor, 40µM), Necroptosis inhibitor Necrostatin1s (Nec1s, 40µM) or Ferroptosis inhibitor Ferrostatin-1 (Fe1, 20µM). ***p ≤ 0.001, Two-Way Anova with multiple comparisons. Values are expressed as mean ± SEM. Graphs show values from three independent experiments with one different donor each time.

•	In lines 220-223, the authors state that P. aeruginosa strains lacking T3SS or flagellin do not drive caspase-1-dependent neutrophil lysis, IL-1b release and PM permeabilization. In order to support this claim, it would be useful to carry out additional statistical analysis comparing WT infected vs WT naïve to show that only the strains lacking T3SS or flagellin prevent neutrophil lysis and not the other mutants, i.e. using a two-way ANOVA.

According to our reviewer suggestions, we have include all suggested statistical comparisons in the revised version of the manuscript.

**Refers to S1B Fig in the revised manuscript.** Measure of cell lysis (release of LDH) and IL-1β release in WT or Casp1-/- murine Bone Marrow Neutrophils (BMNs) infected for 3 hours with Pseudomonas aeruginosa PAO1 and its isogenic mutants lacking T3SS expression (PAO1ΔExsA), Flagellin (PAO1ΔFliC) or T3SS-derived toxins ExoS, ExoY, ExoT (PAO1ΔExoS, PAO1ΔExoY, PAO1ΔExoT) at a multiplicity of infection (MOI) of 10. ***p ≤ 0.001, Two-Way Anova with multiple comparisons. Values are expressed as mean ± SEM. Graphs show combined values from three independent experiments. Values are expressed as mean ± SEM.

In addition, we have performed experiments with *P. aeruginosa* strain PAO1 in which the Flagellin motors have been genetically removed (MotABCD) (doi: 10.1128/IAI.00054-13, doi: 10.1038/s41420-018-0068-z). The generated strains (PAO1^ΔMotABCD^ and PAO1^ΔMotABCD/ΔFliC^) are equally phagocytosed by neutrophils and are known to trigger NLRC4 inflammasome activation in macrophages as they still express non motile flagellin (doi: 10.1128/IAI.00054-13, doi: 10.1038/s41420-018-0068-z). Here, we could show that PAO1^ΔMotABCD^ but not PAO1^ΔMotABCD/ΔFliC^ triggered a Caspase-1-dependent neutrophil pyroptosis and IL-1beta release over time.

As P. aeruginosa strains lacking Flagellin expression or motility, are less efficiently recognized/phagocytosed by immune cells, we did centrifuge both PAO1^ΔMotABCD^ and PAO1^ΔMotABCD/ΔFliC^ on neutrophils to force bacteria-neutrophil contact **(Supplemental Figure 1, below and in manuscript).**

Hence, we are confident that Flagellin plays a major role on the activation of Caspase-1-depndent pyroptosis of neutrophils upon *P. aeruginosa* infection. Those results have been included in Supplemental Figure 1 of the revised manuscript.

**From Supplemental Figure 1. Multiple *P. aeruginosa* strains triggers Caspase-1-dependent pyroptosis in neutrophils. (S1C, S1D in the manuscript)**

Measure of bacterial uptake (Colony-forming Units, CFUs) in WT or *Casp1*^-/-^ BMNs infected for 45 minutes with *Pseudomonas aeruginosa* PAO1 and its isogenic mutants lacking Flagellin (PAO1^ΔFliC^), Flagellin motors MotABCD (PAO1^ΔMotABCD^) or both Flagellin and Flagellin motors MotABCD (PAO1^ΔFliC/ΔMotABCD^) at a MOI of 10. Here, due to their lack of motility, bacteria were centrifuged for 5 min/1000 rpm to ensure neutrophil/bacterial contact. ***p ≤ 0.001, Two-Way Anova with multiple comparisons. Values are expressed as mean ± SEM. Graph show one experiment representative of three independent experiments.

Measure of cell lysis (release of LDH) and IL-1β release in WT and *Casp1*^-/-^ murine Bone Marrow Neutrophils (BMNs) infected for the indicated times with PAO1^ΔMotABCD^ or PAO1^ΔFliC/ΔMotABCD^ at an MOI of 10. Here, due to their lack of motility, bacteria were centrifuged for 5 min/1000 rpm to ensure neutrophil/bacterial contact. ***p ≤ 0.001, Two-Way Anova with multiple comparisons. Values are expressed as mean ± SEM. Graphs show combined values from three independent experiments.

•	The data being discussed in lines 262-265 is shown in Fig 2C and Supp. Figure S2C and not Fig 3E as it is written in the text. It is also not clear why they use the ExoU mutant as the control in these figures rather than the ExoS mutant that is used in the western blot in Fig 2B.

We apologize and have fully corrected all those major issues (mislabeling, wrong referencing, typo mistakes) in our revised manuscript.

•	For several of the experiments, e.g. Fig 2D, Supp. Fig 2C, a naïve control is not used. It would be helpful to include this as a reference point (i.e. to see whether cell death actually significantly differs from the uninfected condition).

Accordingly, we have included all the non-stimulated or uninfected controls in the revised manuscript.

•	The data being discussed in lines 268-275 is shown in Fig 2F and Supp. Figure S2D and not Fig 2D as it is written in the text.

We apologize and have fully corrected all those major issues (mislabeling, wrong referencing, typo mistakes) in our revised manuscript.

•	The data shown in Fig 2D is not discussed in the text despite being an important experiment to show a lack of involvement for the NLRP3 or AIM2 inflammasome in IL-1b release/cell death in response to the P. aeruginosa ExoU mutant.

This is now discussed in the result part as follow:

“We infected WT murine neutrophils or neutrophils from mice lacking expression of various inflammasome sensors, namely Casp1-/-, Casp11-/- , Casp1-/-Casp11-/-, Nlrp3-/-, AIM2-/--, Nlrc4-/- and ASC-/- (S2A Fig.). Among the different tested neutrophil genotypes, significant resistance to pyroptotic cell lysis (LDH release) was only observed in Casp1-/-, Casp1-/-Casp11-/-, Nlrc4-/- and ASC-/- BMNs upon infection with P. aeruginosa pyroptotic strains PAO1ΔExoS and PP34ΔExoU (S2A Fig.). Contrastingly, Casp11-/- , Nlrp3-/- and AIM2-/- neutrophils exhibited similar lysis levels (LDH release) as observed in WT BMNs (S2A Fig.). This suggests that NLRC4 - but not NLRP3, Caspase-11 or AIM2 - efficiently promotes neutrophil pyroptosis upon infection with P. aeruginosa pyroptotic strains (S2A Fig.).”

•	The data shown in Fig 2E is not discussed in the text.

Those results are now discussed in the revised version of the manuscript:

“Further analysis of Caspase-1 (p20) and GSDMD (p30) processing in response to various P. aeruginosa strains showed that the pyroptotic strains PAO1ΔExoS, PP34 ExoUS142A and PP34ΔExoU as well as partially Caspase-1-dependent PAO1 strain triggered robust neutrophil Caspase-1 and GSDMD processing, a process that required NLRC4 expression (Fig. 2B, C). As expected, a PAO1 mutant strain lacking the T3SS regulator ExsA (PAO1ΔExsA) failed to induce robust Caspase-1 and GSDMD processing (Fig. 2B). Finally, the PP34 strain, which promotes NLRC4- and Caspase-1-independent neutrophil lysis, also failed to trigger efficient cleavage of Caspase-1 and GSDMD, suggesting that PP34-induced neutrophil lysis is inflammasome-independent (Fig. 2C). These results suggest that NLRC4 drives Caspase-1 and GSDMD cleavage in response to various P. aeruginosa strains that are capable of inducing neutrophil pyroptosis, namely PAO1, PAO1ΔExoS, CHA, CHAΔExoS, PP34ΔExoU and PP34 ExoUS142A, but not in response to the PP34 strain that induces inflammasome-independent neutrophil lysis.”

•	The data discussed in lines 287-289 refers to the PAO1ExoS- and PP34ExoUS142A strains (not the PAO1ExoU- strain) and this should be edited accordingly in the text.

This has been edited accordingly in the revised version of the manuscript.

•	Lines 294-297 say Fig 3C shows data from pyroptotic strain PP34ExoU- and the NETotic strain PP34ExoU+ but the labels in the figure appear to be different. There also seems to be inconsistencies between text and figure labelling in lines 304-307 and Fig 3D.

We apologize and have fully corrected all those major issues (mislabeling, wrong referencing, typo mistakes) in our revised manuscript.

*****
